# Blood leukocytes as a non-invasive diagnostic tool for thyroid nodules: a prospective cohort study

**DOI:** 10.1186/s12916-024-03368-1

**Published:** 2024-04-02

**Authors:** Feihang Wang, Danyang Zhao, Wang-yang Xu, Yiying Liu, Huiyi Sun, Shanshan Lu, Yuan Ji, Jingjing Jiang, Yi Chen, Qiye He, Chengxiang Gong, Rui Liu, Zhixi Su, Yi Dong, Zhiping Yan, Lingxiao Liu

**Affiliations:** 1 Department of Interventional Radiology, Zhongshan Hospital, Fudan University, Shanghai, 200032 China; 2 National Clinical Research Center for Interventional Medicine, Zhongshan Hospital, Fudan University, Shanghai, 200032 China; 3 Shanghai Institute of Medical Imaging, Shanghai, 200032 China; 4 Department of Ultrasound, Xinhua Hospital Affiliated to Shanghai Jiao Tong University School of Medicine, Shanghai, 200092 China; 5 Singlera Genomics (Shanghai) Ltd., Shanghai, 201203 China; 6 Department of Pathology, Zhongshan Hospital, Fudan University, Shanghai, 200032 China; 7 Department of Endocrinology and Metabolism, Zhongshan Hospital, Fudan University, Shanghai, 200032 China

**Keywords:** Benign thyroid nodule, Blood leukocyte, DNA methylation, Malignant thyroid nodule, Methylation model

## Abstract

**Background:**

Thyroid nodule (TN) patients in China are subject to overdiagnosis and overtreatment. The implementation of existing technologies such as thyroid ultrasonography has indeed contributed to the improved diagnostic accuracy of TNs. However, a significant issue persists, where many patients undergo unnecessary biopsies, and patients with malignant thyroid nodules (MTNs) are advised to undergo surgery therapy.

**Methods:**

This study included a total of 293 patients diagnosed with TNs. Differential methylation haplotype blocks (MHBs) in blood leukocytes between MTNs and benign thyroid nodules (BTNs) were detected using reduced representation bisulfite sequencing (RRBS). Subsequently, an artificial intelligence *b*lood *l*eukocyte *D*NA *m*ethylation (BLDM) model was designed to optimize the management and treatment of patients with TNs for more effective outcomes.

**Results:**

The DNA methylation profiles of peripheral blood leukocytes exhibited distinctions between MTNs and BTNs. The BLDM model we developed for diagnosing TNs achieved an area under the curve (AUC) of 0.858 in the validation cohort and 0.863 in the independent test cohort. Its specificity reached 90.91% and 88.68% in the validation and independent test cohorts, respectively, outperforming the specificity of ultrasonography (43.64% in the validation cohort and 47.17% in the independent test cohort), albeit with a slightly lower sensitivity (83.33% in the validation cohort and 82.86% in the independent test cohort) compared to ultrasonography (97.62% in the validation cohort and 100.00% in the independent test cohort). The BLDM model could correctly identify 89.83% patients whose nodules were suspected malignant by ultrasonography but finally histological benign. In micronodules, the model displayed higher specificity (93.33% in the validation cohort and 92.00% in the independent test cohort) and accuracy (88.24% in the validation cohort and 87.50% in the independent test cohort) for diagnosing TNs. This performance surpassed the specificity and accuracy observed with ultrasonography. A TN diagnostic and treatment framework that prioritizes patients is provided, with fine-needle aspiration (FNA) biopsy performed only on patients with indications of MTNs in both BLDM and ultrasonography results, thus avoiding unnecessary biopsies.

**Conclusions:**

This is the first study to demonstrate the potential of non-invasive blood leukocytes in diagnosing TNs, thereby making TN diagnosis and treatment more efficient in China.

**Supplementary Information:**

The online version contains supplementary material available at 10.1186/s12916-024-03368-1.

## Background

Thyroid nodule (TN) is a widespread occurrence, with a prevalence that can reach as high as 49% [1]. Among these TNs, thyroid cancer (TC) emerges in approximately 7–15% of cases, with papillary thyroid carcinoma (PTC) constituting the majority at around 85% [2]. Individuals harboring palpable TNs or those identified through other imaging methodologies are advised to undergo a thyroid ultrasonography as the first step in risk assessment. This evaluation aids in determining whether a fine needle aspiration (FNA) is warranted [3].

In 2017, the American College of Radiology (ACR) introduced a thyroid imaging reporting and data system (TI-RADS) to systematically assess TNs [4]. This system is extensively employed for thyroid imaging and management. ACR TI-RADS employs a scoring system to classify TNs into five distinct categories, where elevated scores correlate with higher potential for malignancy. Decisions regarding FNA or subsequent monitoring are determined by the risk assessment and the nodule’s maximum diameter. The implementation of ACR TI-RADS has significantly enhanced diagnostic precision while concurrently reducing the need for unnecessary biopsies. Nonetheless, a retrospective study demonstrated that a considerable 57.4% of biopsied were benign thyroid nodules (BTNs) [5]. Another study unveiled that the specificity and positive predictive value (PPV) of ACR TI-RADS 4 and ACR TI-RADS 5 stood at 75% and 47% respectively [6]. Thus, the imperative to devise a new approach aimed at heightening diagnostic efficacy and curbing unnecessary biopsies becomes evident.

In pursuit of early diagnosis and widespread implementation, obtaining blood samples proves to be a less invasive and more convenient alternative compared to tissue samples. Numerous cancer studies have revealed a significant association between cancer risk and DNA methylation in peripheral blood leukocytes, encompassing various types of tumors such as colorectal, bladder, gastric, breast, and head and neck cancers [7–12]. Recently, a diagnostic model for colorectal cancer (CRC) based on five methylation markers in peripheral blood mononuclear cells demonstrated the ability to identify colorectal patients earlier compared to conventional methods [13]. DNA methylation is a well-known stable epigenetic modification that plays a pivotal role in the development of thyroid tumors. Research based on peripheral blood cell-free DNA (cfDNA), such as the study by Shubin Hong et al., has developed a diagnostic tool with 6 markers to distinguish between PTCs and BTNs. When combined with ultrasonography, this approach achieved a sensitivity of 95.7% and a specificity of 70.8% [14]. However, there have been no reported studies on thyroid diagnostic models based on blood leukocytes. Blood leukocytes have a higher abundance and are more convenient for detection compared to blood cfDNA. Leukocyte DNA methylation signatures hold promising applications because they can be non-invasively repeated and are conducive to the dynamic assessment of disease risk. With improved diagnostic accuracy, individuals with BTNs or malignant thyroid nodules (MTNs) can make informed decisions regarding surgery, thermal ablation, or follow-up, taking into account medical recommendations and their own health conditions.

In this study, we developed a *b*lood *l*eukocyte *D*NA *m*ethylation (BLDM) model to assist ultrasonography in improving the specificity and accuracy of distinguishing between MTNs and BTNs, aiding in the selection of patients suitable for further FNA testing, thereby facilitating the triage in the diagnosis and treatment of TNs.

## Methods

### Patient enrollment and sample collection

Between June 2021 and January 2024, a total of 293 peripheral blood samples from treatment-naive patients diagnosed with TNs were collected at the Department of Interventional Radiology, Zhongshan Hospital, Fudan University and Department of Ultrasound, Xinhua Hospital Affiliated to Shanghai Jiao Tong University School of Medicine. The diagnosis of MTN and BTN was confirmed through pathological examination of puncture samples. Following the 2017 World Health Organization classification of endocrine tumors, two experienced pathologists independently evaluated all corresponding sections stained with hematoxylin and eosin as well as immunohistochemical staining sections. The ultrasonography of TNs was evaluated using the ACR-TIRADS. Demographic information, results of laboratory examinations, and ultrasonic imaging findings from the research cohort are displayed in Additional file 1: Table S1. All eligible participants provided consent forms, and this study was approved by the Institutional Review Committee of Zhongshan Hospital, Fudan University (B2022-390R) and Xinhua Hospital Affiliated to Shanghai Jiao Tong University School of Medicine (XHEC-C-2023-028-1).

### Sample size considerations

The formula for estimating sample size at a confidence level of (1-α) % is derived as follows:

$$n=\frac{{\left({Z}_{1-\alpha /2}\right)}^{2}P\left(1-P\right)}{{d}^{2}}$$

Here, *P* represents the predetermined value of sensitivity (or specificity), ${Z}_{1-\alpha /2}$ represents the *z*-value for the standard normal distribution with a left-tail probability (*1-α/2*), and *d* corresponds to half the desired width of the confidence interval [15]. For this study, the objective is to assess sensitivity/specificity at 90% with a 95% confidence level and a maximum marginal error of 0.1. Consequently, a dataset comprising a minimum of 35 malignant thyroid nodules and 35 benign thyroid nodules would be required.

### DNA methylation sequencing and data preprocessing

Approximately 8 ml of venous blood was collected using Streck Cell-Free DNA BCT tubes (xjydna, Fujian, China). To isolate peripheral blood leukocyte, the blood samples were promptly centrifuged twice at 1600 g for 15 min each time at 4 °C. Genomic DNA was then extracted from the peripheral blood leukocyte using QIAamp DNA Mini Kit (Qiagen, Hilden, Germany) following the manufacturer’s instructions. The DNA quantity was measured using the Qubit 4 Fluorometer (ThermoFisher, MA, USA) and the Qubit 1X dsDNA High Sensitivity (HS) Assay Kit (ThermoFisher, MA, USA). All DNA samples were processed using reduced representation bisulfite sequencing (RRBS) at Singlera Genomics (Shanghai) Ltd. In brief, 50 ng of input DNA underwent digestion with MspI and subsequent ligation with a methylated adapter containing a complementary sticky end. The ligated product was subjected to bisulfite conversion and amplification to incorporate Illumina sequencing indices. A specific length range of DNA was selected for sequencing. DNA sequencing was carried out using a NovaSeq 6000 System (Illumina, Inc., CA, USA). Adapters and bases with a quality value < 20 were removed, and reads > 30 base pairs were retained using Trim-Galore (version 0.6.0). Subsequently, the paired-end reads were merged using PEAR (version 0.9.6) with the parameters “-v 20 -n 30” [16] and then aligned to the hg19 genome. The methylation of CpGs was extracted using Bismark (version 1.2.2) [17].

### Identification of differential methylation regions and gene-related annotation

The de novo identification of differential methylation regions (DMRs) between MTN (*n* = 49) vs. BTNs (*n* = 59) within the discovery cohort was carried out using metilene (v0.2–8) [18] with default parameters. *P*-value was adjusted using the false discovery rate (FDR). DMRs meeting the criteria of FDR < 0.05 and |Δβ|≥ 0.02 were selected for gene annotation using the Homer annotatePeaks tool.

### Measurement of methylation haplotype blocks

Methylation haplotype blocks (MHBs), a well-established concept that leverages genetic linkage disequilibrium to assess the degree of co-methylated CpGs, were identified as previously described [19]. We determined methylation levels within an MHB using six types of measurements: average methylation fraction (AMF), methylated haplotype load (MHL), unmethylated haplotype load (UMHL), and proportion of discordant reads (PDR), as well as MHL3 and UMHL3, which assign a different weight corresponding to MHL and UMHL, respectively. The calculation formulae of these measurements have been detailed in our previously published study [20].

### A blood leukocyte DNA methylation model development and validation

Using DNA methylation measurements of MHBs from peripheral blood leukocytes, we developed a diagnostic model to distinguish between MTNs and BTNs. A total of 33,871 MHBs with coverage ≥ 10X and < 500X in ≥ 80% of the samples were identified in the discovery dataset. For each MHB, we selected one measurement type from the six types based on the lowest *P-*value using the Mann–Whitney *U* test between MTNs and BTNs. A total of 70 markers with *P-*value < 0.001 were initially obtained. The selection of the 60 markers was carried out using a Python function called “REFCV”, with the RandomForest estimator and 10-fold cross-validation. The optimal set of markers was determined recursively through cross-validation. Missing values in the discovery data were imputed using the KNNImputer from scikit-learn, with five neighboring samples. Each validated sample had missing values imputed by the same method and was then combined with all discovery samples. The model was trained using the *z*-score standardized features and hyperparameters were determined through tenfold cross-validation on the discovery dataset. A validation cohort consisting of 97 samples and an independent test cohort consisting of 88 samples were used to evaluate the performance of this model. The samples for both the discovery and validation sets were exclusively obtained from the Department of Interventional Radiology, Zhongshan Hospital, Fudan University, amounting to 205 cases. Meanwhile, samples for the independent test cohort were collected from the Department of Ultrasound, Xinhua Hospital Affiliated to Shanghai Jiao Tong University School of Medicine, totaling 88 cases. Sensitivity, specificity, PPV, and negative predictive value (NPV) were calculated using a cutoff value which is determined through three repetitions of fivefold cross-validation on the discovery dataset. The average Youden index from discovery sets is adopted as the model’s cutoff threshold (Additional file 1: Fig. S1).

### Statistical analysis

All statistical analyses were performed using the R software (Version 4.1.1, R Foundation for Statistical Computing, Vienna, Austria). Categorical variables were assessed using Fisher’s exact tests, while continuous variables were analyzed using Kruskal-Wallis test. A significance level of 0.05 was used for statistical significance. A heatmap based on the selected MHB markers was generated using Morpheus, employing hierarchical clustering and the Euclidean distance as a similarity measure. For pathway enrichment analyses, R package clusterProfiler was utilized.

## Results

### Study design and clinical characteristics of participants

**Fig. 1:**
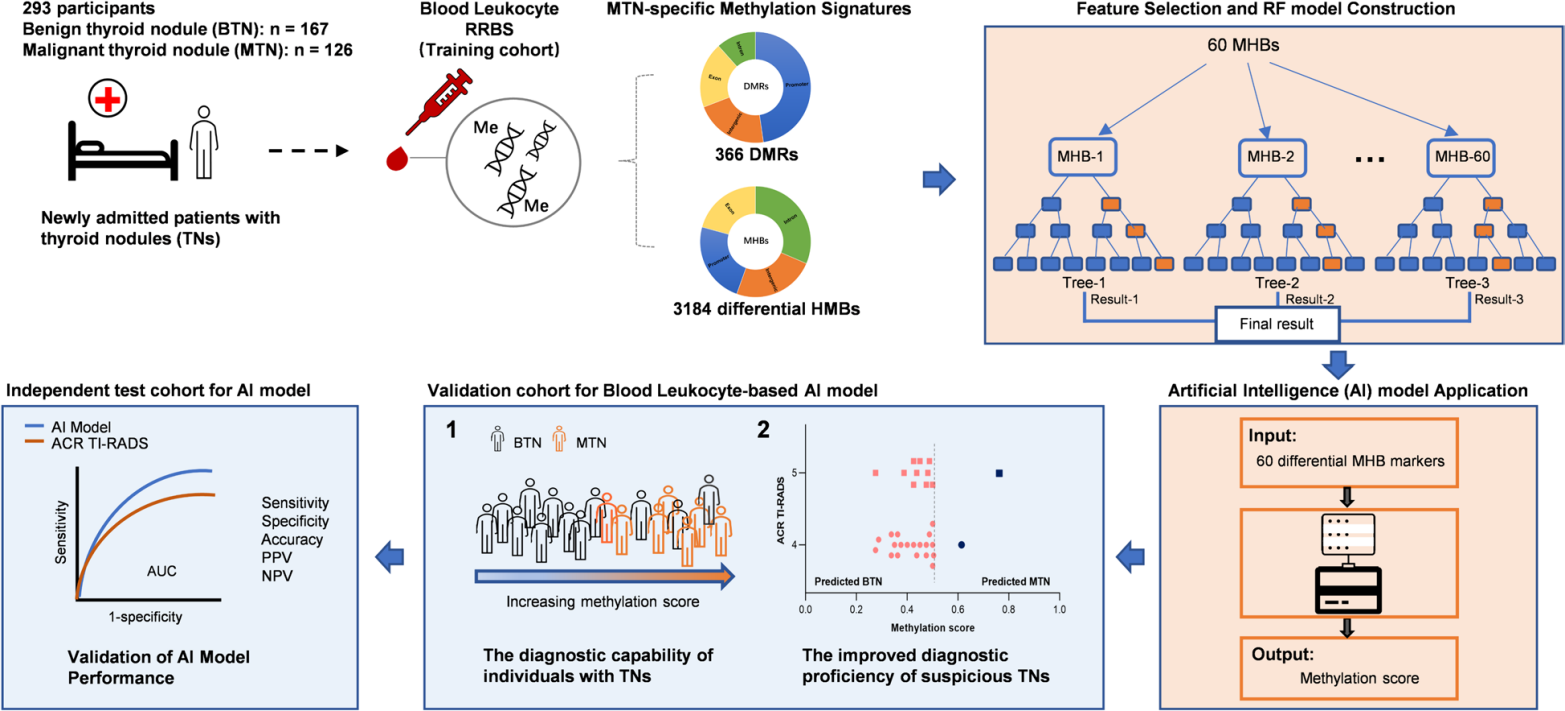
Study design and the workflow of building a BLDM model. Upon patient admission, clinical information was collected, and peripheral blood was obtained for RRBS. A discovery cohort included 59 BTNs and 49 MTNs. Differential leukocyte DNA methylation between MTNs and BTNs were identified, and methylation features were selected to develop a methylation model. The performance of multiple methylation models was compared, and the optimal RF model was selected as the final model and named the BLDM model. The principle of the RF model was shown in the diagram, incorporating a total of 60 MHB biomarkers into the model. The model output methylation scores. The validation cohort comprised 55 cases of BTN and 42 cases of MTN. An independent test cohort consisted of 53 BTNs and 35 MTNs. The performance of the BLDM model was assessed, the correlation between the methylation scores and the benign/malignant nature was analyzed, the performance of the BLDM model in ACR TI-RADS category 4 and 5, and its performance in both micronodules and non-micronodules was assessed. TN, thyroid nodule; BTN, benign thyroid nodule; MTN, malignant thyroid nodule; RRBS, reduced representation bisulfite sequencing; DMR, differential methylation region; MHB, methylation haplotype block; RF, random forest

The study design is depicted in Fig. 1. This study consists of three phases. (1) In the discovery phase, 59 BTN and 49 MTN patients, who had not undergone treatment, were enrolled to assess the genome-wide DNA methylation profiling in peripheral blood leukocyte using RRBS. There were no statistically significant differences in age or sex between patients with MTNs and BTNs (Additional file 1: Table S1). Differential methylation CpGs, regions, and haplotype blocks were identified (Additional file 2). Subsequently, we utilized machine learning algorithms for feature and model selection to construct a diagnostic model for distinguishing MTN and BTN patients. (2) In the validation phase, this model was validated in this cohort comprising 55 BTN and 42 MTN patients, with clinical characteristics similar to the discovery cohort. Specifically, the model’s performance was evaluated in ACR TI-RADS category ≥ 4, as well as in patients with non-micronodules and micronodules, respectively. (3) In the independent test phase, 53 patients with BTNs and 35 patients with MTNs from an external validation cohort were enrolled to further validate the performance of the model. Finally, a TN diagnostic and treatment framework that prioritizes patients is provided.

### Peripheral blood leukocyte DNA methylation differences between MTNs and BTNs

**Fig. 2:**
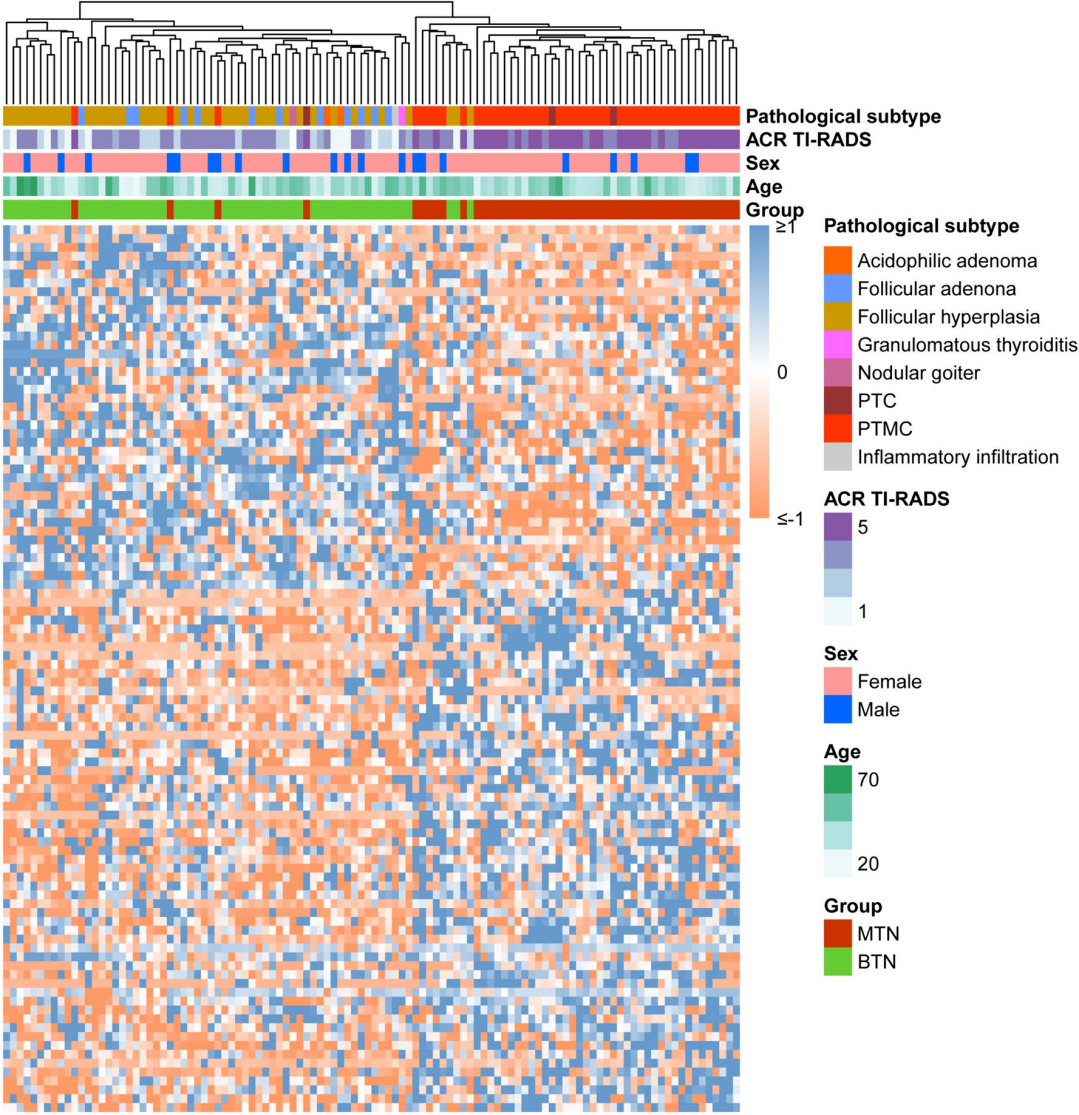
A heatmap displaying top 100 differential MHB markers identified in MTN and BTN groups using *Z*-scores, with clinicopathological details presented on the right side. Significant methylation differences can be observed between the MTN and BTN groups. MHB, methylation haplotype block; BTN, benign thyroid nodule; MTN, malignant thyroid nodule; PTC, papillary thyroid carcinoma; PTMC, papillary thyroid microcarcinoma

For the profiling of DNA methylation in peripheral blood, we employed the RRBS approach, which yielded a substantial number of CpG sites (> 90,000) with a conversion rate exceeding 99%. This method provided highly quantitative methylation data at a base-pair (bp) resolution. The global methylation ratios in peripheral blood showed similar patterns across the two groups (Additional file 1: Fig. S2A). Principal component analysis did not distinguish between MTNs and BTNs, except for the clear distinction based on sex differences, as evident in the first component (Additional file 1: Fig. S2B). We identified 366 DMRs between MTNs and BTNs while controlling FDR at a significance level of 0.05, with a criterion of |Δβ|≥ 0.02. The volcano plot of these DMRs revealed a greater number of hypomethylated regions in MTNs (Additional file 1: Fig. S3A), which aligned with the previous finding [13]. Notably, several genes related to immune response, including fucosyltransferase 4 (*FUT4*) [21], suppressor of cytokine signaling 3 (*SOCS3*) [22], interferon-induced transmembrane protein 1 (*IFITM1*) [23], CD40 molecule (*CD40*) [24], and solute carrier family 7 member 8 (*SLC7A8*) [25], were identified and depicted in the volcano plot (Additional file 1: Fig. S3A). The distribution of gene locations, CpG islands and their shores of these DMRs was represented in pie charts (Additional file 1: Fig. S3B and C). Furthermore, we noted that 6.83% (22 out of 366) of DMRs exhibited overlaps with enhancer regions, each spanning no less than 12 bps, as confirmed through alignment with enhancer database [26]. In investigating differential methylation within MHBs between MTNs and BTNs, we identified a total of 3184 MHBs. The heatmap, which has been scaled by subtracting the mean value and dividing by the standard deviation, showcases the methylation differential trends of top 100 MHBs (Fig. 2). As the differentially MHBs annotated genes did not shed light on the biological mechanisms underlying tumorigenesis, we delved into enriched pathways. We found that MTNs exhibited significant enrichment in calcium signaling pathway (Additional file 1: Fig. S4).

### Development and validation of a TN diagnostic model

Next, we attempt to develop a diagnostic model for distinguishing between MTNs and BTNs by utilizing the well-established MHBs that have already been employed in model construction [20, 27]. We conducted RFECV using a Random Forest model to further refine the marker set. This process resulted in a final set of 60 MHB markers. A heatmap depicts the methylation differences among the two groups for 60 markers (Additional file 1: Fig. S5). Among these markers, 15 were PDR-based markers, 8 AMF-based markers, 10 MHL-based markers, 5 UMHL-based markers, 6 MHL3-based markers, and 16 UMHL3-based markers. These 60 MHB markers were annotated to 58 genes, and notably, 18 of these genes have previously reported to be associated with thyroid dysfunction or thyroid diseases, and 27 were involved in immune regulation (Additional file 1: Table S2 [28–94]).

**Fig. 3:**
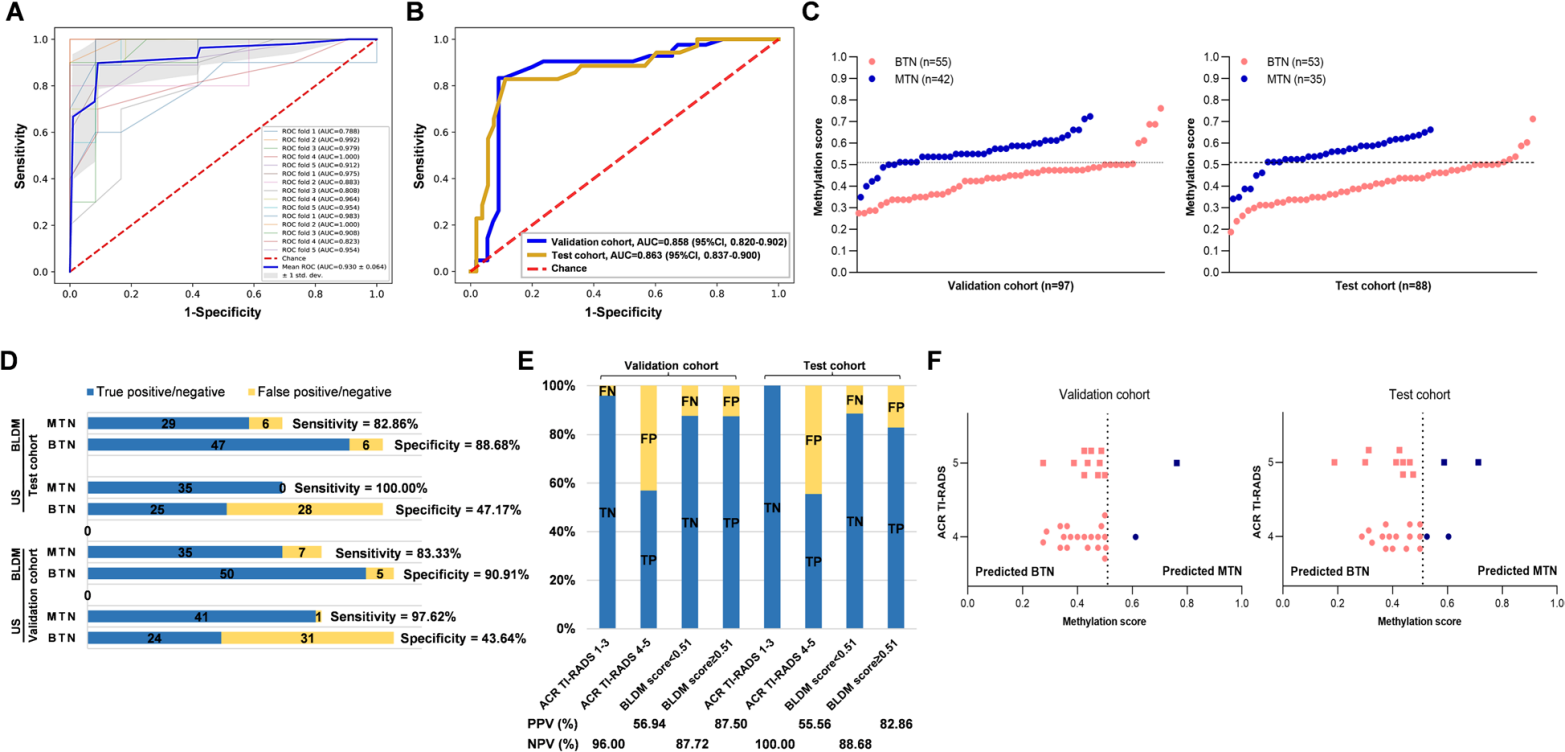
The performance of the BLDM model comparison with ACR TI-RADS. Receiver operating characteristic (ROC) curves of the BLDM model, showcasing its performance in the discovery cohort (**A**), a validation cohort and an independent test cohort, respectively (**B**). The area under the curve (AUC) scores are presented, along with 95% confidence interval values. **C** Methylation scores assigned to TN specimens in the validation cohort and independent test cohort. A threshold of 0.51 was set to distinguish between MTNs and BTNs. **D** A comparison of the sensitivity and specificity of US and the BLDM model in predicting MTNs. **E** The PPV and NPV of US and the BLDM model. Sample numbers in each category are presented at the bottom of the chart. **F** The diagnostic performance of the BLDM model specifically in BTN patients with ACR TI-RADS category ≥ 4 in the validation cohort and independent test cohort. BTN, benign thyroid nodule; MTN, malignant thyroid nodule; TN, thyroid nodule; US, ultrasonography; TP, true positive; FP, false positive; TN, true negative; FN, false negative; PPV, positive predictive value; NPV, negative predictive value

The mean area under the curve (AUC) was 0.930 ± 0.064 (±1 standard deviation) in the three repetitions of the fivefold cross-validation analysis of the discovery data (Fig. 3A). This diagnostic model exhibited a high AUC of 0.858 (95% CI 0.820–0.902) in the validation cohort (Fig. 3B), using a cutoff value of 0.51 determined from the discovery data (Fig. 3C). Based on this criterion, the BLDM model correctly identified 50 out of 55 BTNs as benign, and it accurately detected 35 out of 42 MTNs as malignancy (Additional file 3). While the sensitivity of the BLDM model was lower compared to ultrasonography (83.33% vs. 97.62%), its specificity showed a significant improvement (90.91% vs. 43.64%; Fig. 3D and Table 1). The PPV of the BLDM model outperformed that of ultrasonography (87.50% vs. 56.94%), although the NPV of it was lower compared to ultrasonography (87.72% vs. 96.00%; Fig. 3E).

**Table 1:** Application of the BLDM model and ultrasonography in identifying MTN and BTN samples

(%)	Validation cohort						Independent test cohort					
			> 10 mm		≤ 10 mm				> 10 mm		≤ 10 mm	
	BLDM	US	BLDM	US	BLDM	US	BLDM	US	BLDM	US	BLDM	US
Sensitivity	83.33	97.62	66.67	83.33	86.11	100	82.86	100.00	75.00	100.00	83.87	100.00
Specificity	90.91	43.64	90.00	57.50	93.33	6.67	88.68	47.17	85.71	67.86	92.00	24.00
Accuracy	87.63	67.01	86.96	60.87	88.24	72.55	86.36	68.18	84.38	71.88	87.50	66.07
PPV	87.50	56.94	50.00	22.73	96.88	72.00	82.86	55.56	42.86	30.77	92.86	62.00
NPV	87.72	96.00	94.74	95.83	73.68	100.00	88.68	100.00	96.00	100.00	82.14	100.00
AUC	0.86	0.85	0.87	0.87	0.86	0.65	0.86	0.82	0.87	0.98	0.86	0.68

*MTN* Malignant thyroid nodule, *BTN* Benign thyroid nodule, *US* Ultrasonography, *PPV* Positive predictive value, *NPV* Negative predictive value, *AUC* Area under the curve

### Independent test of the BLDM model to distinguish MTNs and BTNs

The BLDM demonstrated an AUC of 0.863 (95% CI 0.837–0.900) in the independent test cohort (Fig. 3B). It correctly identified 47 out of 53 BTNs as benign and accurately recognized 29 out of 35 MTNs as malignant (Additional file 4). Compared to ultrasonography (sensitivity: 100.00%; specificity: 47.17%), the sensitivity of the BLDM model was lower (82.86%), while its specificity showed a significant improvement (88.68%) (Fig. 3D and Table 1). The PPV of the BLDM model outperformed that of ultrasonography (82.86% vs. 55.56%), although the NPV of it was lower compared to ultrasonography (88.68% vs. 100.00%) (Fig. 3E). Therefore, the BLDM model exhibits substantial potential as a diagnostic tool for TNs in specimens displaying distinctive epigenetic signatures.

### The BLDM model for enhanced diagnosis of suspicious TNs

In the validation and independent tests, there were initially 20 and 17 patients presenting moderately suspicious MTNs, respectively, categorized as ACR TI-RADS category 4. Additionally, in both cohorts, 11 patients with highly suspicious MTNs classified under ACR TI-RADS category 5 were all histologically confirmed as benign following core needle biopsy. Remarkably, employing our model, 19 out of 20 cases (95.00%) within ACR TI-RADS 4 and 10 out of 11 cases (90.91%) in ACR TI-RADS 5 were accurately predicted as benign in the validation cohort (Fig. 3F). In the independent test cohort, our model accurately predicted 15 out of 17 benign cases (88.24%) within ACR TI-RADS 4 and correctly identified 9 out of 11 benign cases (81.82%) in ACR TI-RADS 5. This achievement reflects the impressive accuracy of the model, reaching 89.83%.

### The performance of the BLDM model in ACR TI-RADS categories 4 and 5

In the context of ACR TI-RADS ≥ 4, the BLDM model exhibited an impressive AUC of 0.869 (95% CI: 0.769–0.968) and 0.822 (95% CI: 0.710–0.935) in the validation cohort and independent test cohort, respectively, surpassing that of ultrasonography. The sensitivity, specificity, and accuracy of the BLDM model in the validation cohort reached noteworthy levels at 88.57%, 93.55%, and 90.91%, respectively (Additional file 1: Fig. S6A). Furthermore, within the ACR TI-RADS 4 subgroup, the BLDM model performed exceptionally well, achieving an AUC of 0.910 (95% CI: 0.786–1.000). Comparatively, the BLDM model displayed a higher specificity, accuracy, and NPV in ACR TI-RADS 4, surpassing ACR TI-RADS 5 (Additional file 1: Fig. S6B and C). In the independent test cohort, the BLDM model achieved impressive sensitivity, specificity, and accuracy levels of 82.86%, 85.71%, and 84.13%, respectively (Additional file 1: Fig. S6D). Within the ACR TI-RADS 4 subgroup, the BLDM model obtained an AUC of 0.843 (95% CI: 0.676–1.000). In ACR TI-RADS 5, the BLDM model attained an AUC of 0.783 (95% CI: 0.583–0.982). Similarly, the BLDM model demonstrated higher sensitivity, specificity, accuracy, and NPV in the ACR TI-RADS 4 category (Additional file 1: Fig. S6E and F).

### Comparison of the BLDM model and ACR TI-RADS for detecting non-micronodules

**Fig. 4:**
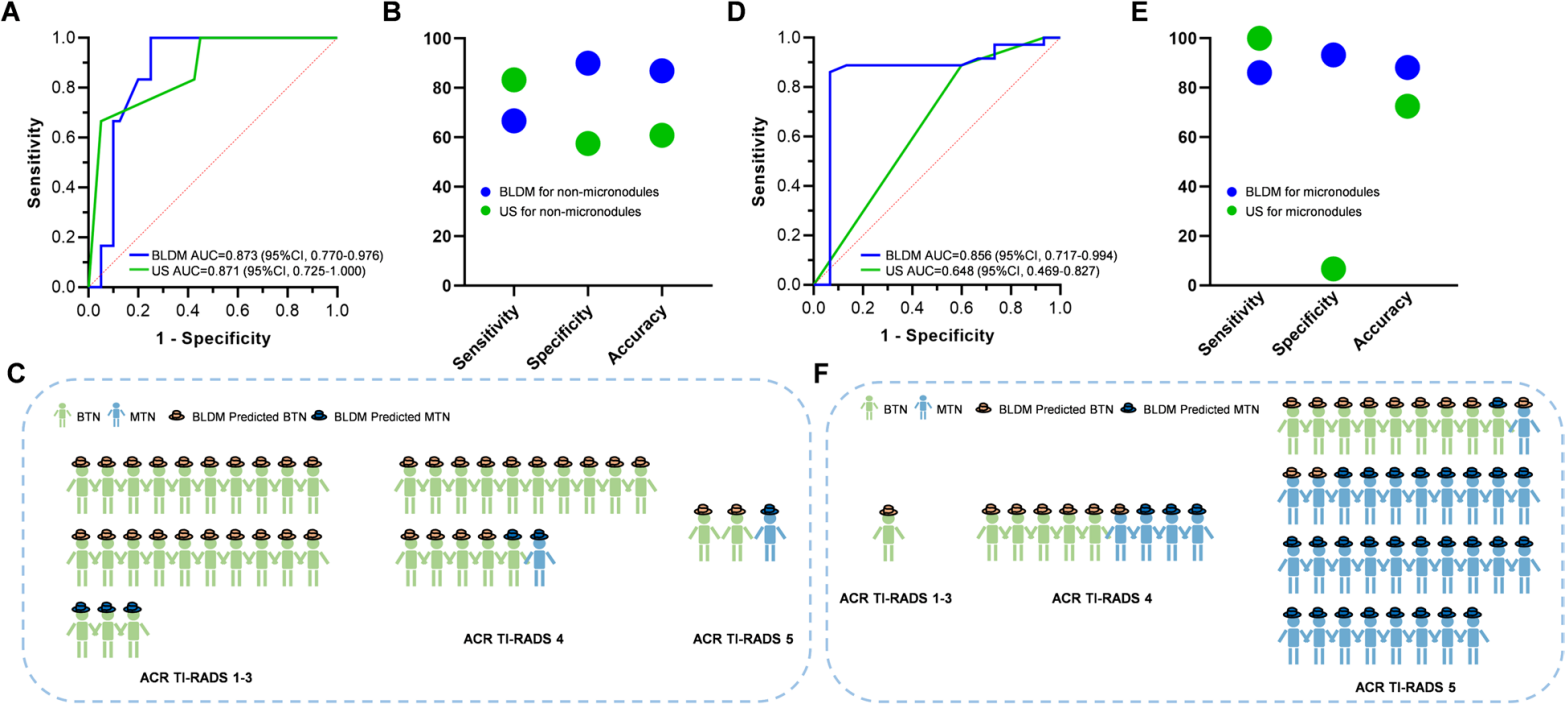
Performance of the BLDM model in classifying MTN and BTN samples in both non-micronodules and micronodules within the validation cohort. **A** Area under the curve (AUC) scores of the BLDM model and US for non-micronodules. **B** Comparing the performance of the BLDM model and US in non-micronodules. **C** The diagnostic performance of the BLDM model for non-micronodules across different ACT TI-RADS categories. **D** AUC scores of the BLDM model and US for micronodules. **E** Comparing the performance of the BLDM model and US in micronodules. **F** The diagnostic performance of the BLDM model for micronodules across different ACT TI-RADS categories. BTN, benign thyroid nodule; MTN, malignant thyroid nodule; US, ultrasonography

The BLDM model demonstrated an AUC of 0.873 (95% CI: 0.770–0.976), similar to ACR TI-RADS (Fig. 4A), while maintaining notably higher specificity and accuracy compared to ACR TI-RADS (specificity: 90.00% vs. 57.50%; accuracy: 86.96% vs. 60.87%) in the validation cohort. In contrast, ACR TI-RADS achieved a higher sensitivity of 83.33% compared to BLDM’s 66.67% (Fig. 4B and Table 1). In the independent test cohort, the BLDM model demonstrated an AUC of 0.866 (95% CI: 0.694–1.000) (Additional file 1: Fig. S7A), featuring a specificity of 85.71% and an accuracy of 84.38%, consistent with its performance in the validation cohort, outperforming ACR TI-RADS. Additionally, the sensitivity of BLDM was lower than that of ACR TI-RADS (Table 1 and Additional file 1: Fig. S7B). Among non-micronodules in the validation cohort, 23 patients were categorized within ACR TI-RADS categories 1–3, and the BLDM model accurately predicted 20 as benign. On the other hand, there were 15 cases with ACR TI-RADS category 4, but they were pathologically diagnosed as benign. In this scenario, the model correctly identified the vast majority (14 out of 15 cases) of benign patients. Among the three patients classified as ACR TI-RADS 5, two of them were BTNs, and the model made correct predictions for both (Fig. 4C). Among the 8 cases categorized as ACR TI-RADS 4 but pathologically confirmed as benign in the independent test cohort, the model correctly identified 75.00% (6/8) of the BTNs. In the 3 cases classified as ACR TI-RADS 5, one patient was pathologically confirmed as benign, and the model accurately identified this case (Additional file 1: Fig. S7C).

### The BLDM model effectively distinguished papillary thyroid microcarcinoma (PTMC) from benign micronodules

Considering the limitations in the diagnostic performance of micronodules using ultrasonography, we conducted further investigations to determine whether our model could effectively distinguish PTMC from BTN (≤ 10 mm). Remarkably, our model demonstrated exceptional discriminatory capabilities, achieving an AUC of 0.856 (95% CI: 0.717–0.994) in the validation cohort and 0.858 (95% CI: 0.794–0.968) in the independent test cohort (Fig. 4D and Additional file 1: Fig. S7D). In contrast, the AUC for ACR TI-RADS was 0.648 (95% CI: 0.469–0.827) in the validation cohort and 0.677 (95% CI: 0.533–0.822) in the independent test cohort. The sensitivity of ACR TI-RADS was higher than that of the BLDM model (100% vs. 86.11% in the validation cohort and 100% vs. 83.87% in the independent test cohort), while its specificity and accuracy were lower than that of BLDM (specificity: 6.67% vs. 93.33% in the validation cohort and 24.00% vs. 92.00% in the independent test cohort; accuracy: 72.55% vs. 88.24% in the validation cohort and 66.07% vs. 87.50% in the independent test cohort; Fig. 4E and Additional file 1: Fig. S7E). Among patients with micronodules, the BLDM model correctly classified all patients with ACR TI-RADS 4 as pathologically benign (5/5), and the vast majority of patients with ACR TI-RADS 5 as pathologically benign (8/9) in the validation cohort (Fig. 4F). In the independent validation cohort, the BLDM model consistently identified all patients with pathologically confirmed BTNs in the ACR TI-RADS 4 and 80.00% (8/10) of pathologically confirmed BTNs in ACR TI-RADS 5 category (Additional file 1: Fig. S7F). The BLDM model exhibits higher sensitivity, specificity, accuracy, and PPV compared to its performance in non-micronodules, highlighting its superiority in micronodule patients.

**Fig. 5:**
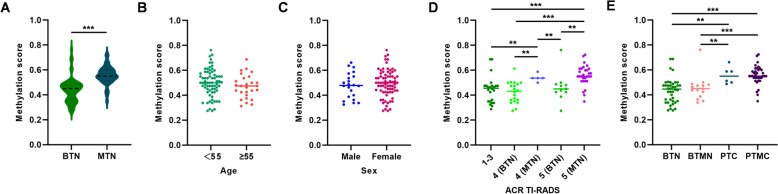
Association of clinical features and methylation scores in the validation cohort. The scatter plots depict methylation scores in relation to pathology (**A**), age (**B**), gender (**C**), ACR TI-RADS (**D**), and nodule sizes (**E**). The black horizontal line represents the median methylation levels. BTN, benign thyroid nodule; MTN, malignant thyroid nodule; BTMN, benign thyroid micronodule; PTC, papillary thyroid carcinoma; PTMC, papillary thyroid microcarcinoma. **P < 0.01, ***P < 0.001

Additionally, we observed that methylation scores were only associated with malignancy status (Fig. 5A and Additional file 1: Fig. S8A), with no significant correlations observed with age, sex, ACR TI-RADS category, and nodule size (Fig. 5B–E and Additional file 1: Fig. S8B–E).

### Application of the BLDM model in the diagnosis and treatment of TNs

**Fig. 6:**
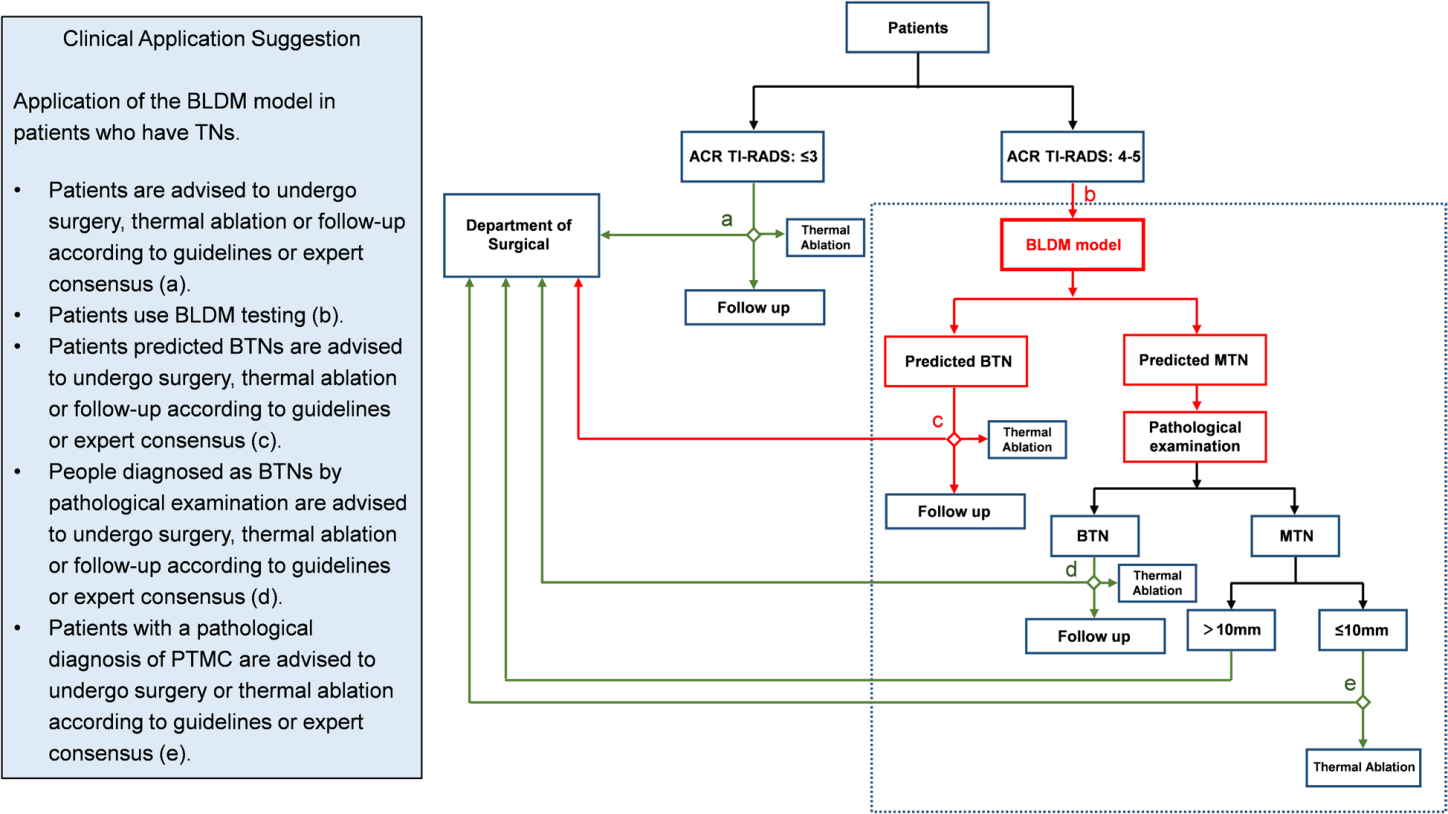
Patient examination and treatment flowchart. 1. Patient admission; 2. Ultrasonography examination and ACR TI-RADS classification; 3. Management recommendations (ACR TI-RADS 1–3) or blood tests (ACR TI-RADS 4–5) according to ACR TI-RADS categories; 4. Management recommendations (BLDM predicted BTNs) or pathological examination (BLDM predicted MTNs) according to methylation scores. The red lines represent the clinical application of the BLDM model, while the green lines represent the patient management recommended by guidelines or expert consensus. TN, thyroid nodule; BTN, benign thyroid nodule; MTN, malignant thyroid nodule; PTMC, papillary thyroid microcarcinoma

All patients with TNs who visited the hospital underwent ultrasonography examinations, which encompassed various parameters. Patients falling under ACR TI-RADS categories 1–3 were recommended for surgery, thermal ablation, or follow-up in accordance with guidelines [95–97]. On the other hand, patients categorized as ACR TI-RADS 4–5 were recommended to undergo blood tests. The BLDM predicted results indicated that patients predicted to have BTNs followed the same treatment process as ACR TI-RADS 1–3 category patients. Patients predicted to have MTNs according to BLDM were advised to undergo cytological examination. Cytological examination for patients with BTNs followed the recommended guidelines for surgery, thermal ablation, or follow-up. For patients with MTNs identified through cytological examination, the choice of treatment, including thermal ablation, was based on factors such as nodule size and malignancy (Fig. 6). Incorporating blood BLDM examinations can help avoid unnecessary FNA biopsies, and it can offer a more rational and efficient treatment approach for TN patients.

## Discussion

The diagnosis of thyroid cancer has long presented challenges because of the overlapping ultrasonography features seen in both BTNs and MTNs. Although various thyroid imaging systems have undeniably improved the accuracy of diagnostic evaluations, there is still room for further improvement in their performance. For a considerable duration, the implementation of ACR TI-RADS has indeed contributed to the improved diagnostic accuracy of TNs. However, a significant issue persists where many patients undergo unnecessary biopsies, primarily due to the relatively low specificity of ACR TI-RADS categories 4 and 5. A meta-analysis conducted by Kang et al. demonstrated that the sensitivity of ACR TI-RADS 4 reached 94.37%, but its specificity was notably low, standing at only 52.24%. In comparison, the specificity of ACR TI-RADS 5 was better at 86.96%, surpassing TR4 [98]. Furthermore, another prospective study that compared the diagnostic performance of ACR TI-RADS, K-TIRADS, and ATA guidelines revealed that the specificity of ACR TI-RADS 4/5 was only 66.3%, with a PPV of 30.6%. Notably, the rate of unnecessary FNA for cases classified under ACR TI-RADS was as high as 32.0% [99]. There are very few diagnostic models based on blood leukocytes. However, blood leukocytes not only provide the advantage of convenient and swift sampling but are also highly adaptable to testing. In this context, we have introduced an innovative approach centered on peripheral blood leukocyte DNA methylation to differentiate between MTNs and BTNs. In our study, the BLDM model exhibited superior specificity when compared to ACR TI-RADS, with a specificity of 90.91% versus 43.64% in the validation cohort and a specificity of 88.68% versus 47.17% in the independent test cohort. This superior performance was particularly notable in ACR TI-RADS 4 and ACR TI-RADS 5 categories. This enhanced diagnostic capability of the BLDM model effectively identified cases of BTNs initially suspected as thyroid cancer based on ultrasonography.

Furthermore, for micronodules, the diagnostic performance of ACR TI-RADS had a sensitivity of 78.3%, specificity of 57.1%, and overall accuracy of 73.9%. These findings were consistent with those of another study by Qi et al., which assessed the diagnostic efficacy of five different systems (C-TIRADS, ACR TI-RADS, Kwak-TIRADS, KSThR-TIRADS, and EU-TIRADS) using a total of 1096 nodules (682 benign and 414 malignant). For micronodules, ACR TI-RADS demonstrated a sensitivity of 70.4%, specificity of 68.4%, PPV of 53.2%, and an accuracy of 69.1%. In comparison, EU-TIRADS exhibited values of 77.5% for sensitivity, 60.4% for specificity, 50.0% for PPV, and 66.2% for accuracy, respectively [100]. In our study, the BLDM model exhibited a strong capacity to differentiate between PTMC and micronodular BTN. It achieved a sensitivity of 86.11%, specificity of 93.33%, and an overall accuracy of 88.24% in the validation cohort and a sensitivity of 83.87%, specificity of 92.00%, and accuracy of 87.50% in the independent test cohort. All of these confirm that the application of the BLDM model has the potential to significantly improve diagnostic accuracy, particularly in patients with indeterminate TNs on ultrasonography and in the case of micronodules, with the potential to reduce unnecessary biopsies. Worth mentioning is, in both the validation and independent test cohorts, the percentage of MTNs in patients with nodules ≤ 10 mm (micronodules) was higher (36/51 cases, 70.59% and 31/56 cases, 55.36%) compared to the proportion of MTNs in patients with TNs larger than 10 mm (non-micronodules) (6/36 cases, 13.04% and 4/32 cases, 12.50%). This situation arises due to the majority of patients with micronodules enrolled in the department of interventional radiology had ACR TI-RADS grades of 4–5. This highlights that our model effectively addresses the challenge of low ultrasonography specificity in TN patients, particularly those with micronodules (ACR TI-RADS 4–5 micronodules). Leveraging the excellent diagnostic performance of the BLDM model can facilitate early diagnosis and non-invasive monitoring for individuals with TNs. This offers them the option to undergo minimally invasive treatments. It is crucial to emphasize that the model is not intended to replace ultrasonography or FNA but rather to reduce the necessity for unnecessary biopsies.

Blood leukocytes play a crucial role in the immune system, participating in various aspects of the immune signaling pathways. This includes the presence of multiple receptors on the surface of blood leukocytes, such as T cell receptors, B cell receptors, and antigen-presenting receptors [101, 102]. These receptors are responsible for recognizing foreign antigens and triggering immune signal transduction pathways. Blood leukocytes also play a significant role in inflammation response and immune cell activation [103–105]. Methylation can influence the expression of immune-related genes in leukocytes, including antigen-presenting genes, immune checkpoint genes, and effector molecules of cytotoxic T cells, with profound implications for immune evasion, and immune suppression [106, 107]. Furthermore, this study revealed that the calcium signaling pathway is the most significantly enriched pathway. Calcium signaling plays a crucial role in B cell development and can be intricately regulated through B cell receptor (BCR)-dependent pathways, which significantly contribute to the mechanisms that maintain self-tolerance. Numerous studies have established a link between the disruption of calcium signaling and the breakdown of tolerance, which ultimately leads to the development of autoimmunity in genetically modified mouse strains [108, 109]. The role of altered calcium flux in B cells has also been discussed in the context of thyroid autoimmunity in a prior study [110]. These findings may provide an explanation for why the calcium signaling pathway emerges as the most prominent pathway in MTN patients. Does the origin of DNA methylation markers in the diagnostic model lie exclusively with B cells? This will be further investigated in our subsequent research to explore the source and mechanisms of these markers. In this study, the BLDM model is designed for distinguishing between MTNs and BTNs and is not suitable for general health screening in the population. In subsequent studies, we will incorporate samples from healthy individuals to develop a more convenient and non-invasive method for TN screening.

## Conclusions

This is the first study to demonstrate that blood leukocyte DNA methylation is a non-invasive diagnostic tool for TNs, thus reducing the need for unnecessary FNA. Our research findings indicate that blood leukocyte DNA methylation is well-suited for TN diagnosis when ultrasonography is inconclusive, aiding in patient triage. Therefore, we propose a thyroid nodule diagnostic and treatment framework, offering more treatment options for certain BTN patients and those with microcarcinomas. We aim to improve the current issue of overdiagnosis and overtreatment of TNs in China, making the diagnosis and treatment process for TNs more rational and efficient.

### Supplementary Information

**Additional file 1: Figure S1.** Flow chart of AI model establishment. **Figure S2.** Epigenetic alterations identified in the discovery cohort. **Figure S3.** Differentially methylated regions (DMRs) between MTNs (*n* = 59) and BTNs (*n* = 49). **Figure S4.** KEGG pathways significantly enriched for MHBs. **Figure S5.** A heatmap displaying 60 MHB markers involved in the model, with clinicopathological details presented on the right side. **Figure S6.** Performance of the BLDM model in classifying MTN and BTN samples in ACR TI-RADS category 4 and 5. **Figure S7.** Performance of the BLDM model in classifying MTN and BTN samples in both non-micronodules and micronodules within the independent test cohort. **Figure S8.** Association of clinical features and methylation scores in the independent test cohort. **Table S1.** Comparison of clinicopathological characteristics between benign and malignant nodules. **Table S2.** The role of 60 MHB markers involved in the BLDM model. **Table S3.** Clinicopathological characteristics of non-micronodules and micronodules in the study cohort.

**Additional file 2.**

**Additional file 3.**

**Additional file 4.**

## Data Availability

The datasets used and/or analyzed during the current study are available from the corresponding author on reasonable request.

## Abbreviations

ACR: The American College of Radiology
AMF: Average methylation fraction
AUC: Area under the curve
BCR: B cell receptor
BLDM: Blood leukocyte DNA methylation model
BP: Base-pair
BTN: Benign thyroid nodule
CD40: CD40 molecule
cfDNA: Cell-free DNA
CRC: Colorectal cancer
DMR: Differential methylation region
FDR: False discovery rate
FNA: Fine needle aspiration
FUT4: Fucosyltransferase 4
IFITM1: Interferon-induced transmembrane protein 1
MHB: Methylation haplotype block
MHL: Methylated haplotype load
MTN: Malignant thyroid nodule
NPV: Negative predictive value
PDR: Proportion of discordant read
PPV: Positive predictive value
PTC: Papillary thyroid carcinoma
PTMC: Papillary thyroid microcarcinoma
RFECV: Recursive feature elimination with cross-validation
RRBS: Reduced representation bisulfite sequencing
SLC7A8: Solute carrier family 7 member 8
SOCS3: Suppressor of cytokine signaling 3
TC: Thyroid cancer
TI-RADS: Thyroid imaging reporting and data system
TN: Thyroid nodule
UMHL: Unmethylated haplotype load
