# Supplementary material for: Blood leukocytes as a non-invasive diagnostic tool for thyroid nodules: a prospective cohort study
Source: BMC Med. 2024 Apr 2;22:147. doi: 10.1186/s12916-024-03368-1 (PMC10986011; doi:10.1186/s12916-024-03368-1)
Supplement: Supplementary file 1 — Additional file 1: Figure S1. Flow chart of AI model establishment. Figure S2. Epigenetic alterations identified in the discovery cohort. Figure S3. Differentially methylated regions (DMRs) between MTNs (n = 59) and BTNs (n = 49). Figure S4. KEGG pathways significantly enriched for MHBs. Figure S5. A heatmap displaying 60 MHB markers involved in the model, with clinicopathological details presented on the right side. Figure S6. Performance of the BLDM model in classifying MTN and BTN samples in ACR TI-RADS category 4 and 5. Figure S7. Performance of the BLDM model in classifying MTN and BTN samples in both non-micronodules and micronodules within the independent test cohort. Figure S8. Association of clinical features and methylation scores in the independent test cohort. Table S1. Comparison of clinicopathological characteristics between benign and malignant nodules. Table S2. The role of 60 MHB markers involved in the BLDM model. Table S3. Clinicopathological characteristics of non-micronodules and micronodules in the study cohort. [file 12916_2024_3368_MOESM1_ESM.docx]

Supplementary Table 1. Comparison of clinicopathological characteristics between benign and malignant nodules

|  | Discovery cohort | | Validation cohort | | Test cohort | |
| --- | --- | --- | --- | --- | --- | --- |
| Characteristics | Benign nodules (n=59) | Malignant nodules (n=49) | Benign nodules (n=55) | Malignant nodules (n=42) | Benign nodules (n=53) | Malignant nodules (n=35) |
| Age, years |  |  |  |  |  |  |
| Median (range) | 47 (20, 78) | 44 (22, 70) | 47 (25, 87) | 40 (25, 76) | 51 (19, 74) | 40 (17, 74) |
| Sex, n (%) |  |  |  |  |  |  |
| Male | 11 (18.6) | 10 (20.4) | 12 (21.8) | 10 (23.8) | 16 (30.2) | 15 (42.9) |
| Female | 48 (81.4) | 39 (79.6) | 43 (78.2) | 32 (76.2) | 37 (69.8) | 20 (57.1) |
| ACR TI-RADS, n (%) |  |  |  |  |  |  |
| 1-3 | 30 (50.8) | 0 (0.0) | 24 (43.6) | 0 (0.0) | 25 (47.2) | 0 (0.0) |
| 4 | 29 (49.2) | 14 (28.6) | 20 (36.4) | 5 (11.9) | 17 (32.1) | 12 (34.3) |
| 5 | 0 (0.0) | 35 (71.4) | 11 (20.0) | 30 (71.4) | 11 (20.7) | 23 (65.7) |
| Not graded |  |  | 0 (0.0) | 7 (16.7) |  |  |
| Diameter, mm |  |  |  |  |  |  |
| Average (range) | 21.5 (3.3, 51.3) | 5.2 (2.7,12.0) | 20.1 (3.3, 53.0) | 7.4 (3.3, 46.5) | 17.5 (2.7, 59.0) | 5.6 (2.3, 11.7) |
| Micronodule, n (%) |  |  |  |  |  |  |
| Yes | 13 (22.0) | 46 (93.9) | 15 (27.3) | 36 (85.7) | 25 (47.2) | 31 (88.6) |
| No | 46 (78.0) | 3 (6.1) | 40 (72.7) | 6 (14.3) | 28 (52.8) | 4 (11.4) |
| Lymph node metastasis, n (%) |  |  |  |  |  |  |
| Yes | 0 (0.0) | 0 (0.0) | 0 (0.0) | 7 (16.7) | 0 (0.0) | 0 (0.0) |
| No | 59 (100.0) | 49 (100.0) | 55 (100.0) | 35 (83.3) | 0 (0.0) | 0 (0.0) |

A micronodule is defined as a nodule with a diameter of 10 mm or smaller.

Supplementary Table 2. The role of 60 MHB markers involved in the BLDM model

| **Markers** | **Feature Importance** | **Gene Name** | **Gene Description** | **Functions** | **Thyroid functions** | **Immune functions** |
| --- | --- | --- | --- | --- | --- | --- |
| chr5:72598984:72598989_UMHL3 | 0.067 | LINC02230 | long intergenic non-protein coding RNA 2230 | / |  |  |
| chr3:96495744:96495785_UMHL | 0.057 | EPHA6 | EPH receptor A6 | Involved in vascular and axon guidance | No | No |
| chr5:42924409:42924459_AMF | 0.033 | FLJ32255 | uncharacterized LOC643977 | Associated with melatonin levels | No | No |
| chr9:114245252:114245260_AMF | 0.033 | ECPAS | Ecm29 proteasome adaptor and scaffold | Involved in ubiquitin-dependent ERAD pathway | No | No |
| chr10:134600258:134600302_MHL | 0.032 | NKX6-2 | NK6 homeobox 2 | Linked to spastic ataxia 8 with hypomyelinating leukodystrophy | No | No |
| chr7:4922765:4922802_PDR | 0.028 | RADIL | Rap associating with DIL domain | Regulate Ras signaling, cell adhesion, and the epithelial-mesenchymal transition | No | No |
| chr2:232791101:232791148_AMF | 0.026 | NPPC | natriuretic peptide C | Regulates vital physiological functions in the cardiovascular system | No | No |
| chr1:202776149:202776284_MHL3 | 0.026 | KDM5B | lysine demethylase 5B | Associated with the proliferation inhibition and apoptosis promotion of PTC cells / Promotes immune evasion by recruiting SETDB1 to silence retroelements | Yes | Yes |
| chr7:27212883:27213062_MHL | 0.025 | HOXA10 | homeobox A10 | Regulates PTC and ATC initiation and progression / Associated with immune suppression in PDA | Yes | Yes |
| chr10:114594519:114594642_AMF | 0.023 | LOC103344931 | uncharacterized LOC103344931 | / |  |  |
| chr6:27228227:27228268_PDR | 0.023 | PRSS16 | serine protease 16 | Plays a role in the alternative antigen presenting pathway used by cortical thymic epithelial cells during the positive selection of T cells | No | Yes |
| chr15:76633785:76634041_MHL3 | 0.022 | ISL2 | ISL LIM homeobox 2 | Regulates the transcriptional levels of marker genes in hypothalamus-pituitary-thyroid axis | Yes | No |
| chr11:134341177:134341187_PDR | 0.022 | B3GAT1-DT | B3GAT1 divergent transcript | / |  |  |
| chr2:162930212:162930284_MHL | 0.021 | DPP4 | dipeptidyl peptidase 4 | A potential prognostic marker of thyroid carcinoma and a target for immunotherapy / Expresses in various immune cells and regulates their functions | Yes | Yes |
| chr12:125000613:125000883_PDR | 0.021 | NCOR2 | nuclear receptor corepressor 2 | Circular RNA circ-NCOR2 accelerates PTC progression / Correlated with the infiltrations of various immune signatures | Yes | Yes |
| chr10:135178449:135178609_UMHL3 | 0.019 | MIR3944 | microRNA 3944 | Involved in drug refractory dilated cardiomyopathy | No | No |
| chr19:8174155:8174267_UMHL3 | 0.019 | FBN3 | fibrillin 3 | Involved in the pathogenesis of HT, and polycystic ovary syndrome | Yes | No |
| chr9:94712520:94712581_UMHL3 | 0.019 | ROR2 | receptor tyrosine kinase like orphan receptor 2 | Associated with the disease progress and poor prognosis of thyroid tumors | Yes | No |
| chr10:71122474:71122535_MHL3 | 0.018 | HK1 | hexokinase 1 | The expression of HK1 was associated with PET positive and (18) FDG uptake | Yes | No |
| chr7:27215299:27215672_UMHL | 0.018 | HOXA10 | homeobox A10 | Regulates PTC and ATC initiation and progression | Yes | No |
| chr1:9903073:9903206_MHL | 0.018 | CLSTN1 | calsyntenin 1 | Regulates axon branching and endosomal trafficking | No | No |
| chr8:11445789:11445888_UMHL3 | 0.017 | LINC00208 | long intergenic non-protein coding RNA 208 | Associated with either Barrett’s oesophagus or oesophageal adenocarcinoma | No | No |
| chr10:77871851:77871936_PDR | 0.017 | LRMDA | leucine rich melanocyte differentiation associated | A prognostic signature for PDA | No | No |
| chr8:24813967:24813984_PDR | 0.017 | NEFL | neurofilament light chain | An immune-related gene in diseases | No | Yes |
| chr18:77251761:77251776_MHL | 0.017 | LOC284240 | uncharacterized LOC284240 | / |  |  |
| chr20:33146070:33146819_UMHL | 0.016 | MAP1LC3A | microtubule associated protein 1 light chain 3 alpha | High expression in PTC and metastatic lymph nodes / Associated with immune microenvironment in CRC | Yes | Yes |
| chr22:50473587:50473604_PDR | 0.016 | / |  | / |  |  |
| chr14:21269942:21270203_PDR | 0.015 | RNASE1 | ribonuclease A family member 1, pancreatic | Regulates hemostasis, inflammation, and innate immunity | No | Yes |
| chr10:90846868:90847011_UMHL | 0.015 | MIR4679-2 | microRNA 4679-2 | / |  |  |
| chr10:130182493:130182560_UMHL3 | 0.015 | LINC01163 | long intergenic non-protein coding RNA 1163 | / |  |  |
| chr22:49081733:49081764_UMHL3 | 0.014 | MIR4535 | microRNA 4535 | A diagnostic marker for foetal morbidity of infection | No | No |
| chr1:42385756:42385944_PDR | 0.013 | HIVEP3 | HIVEP zinc finger 3 | Inhibits fate decision of CD8+ invariant NKT cells | No | Yes |
| chr17:85092:85115_UMHL3 | 0.013 | LINC02091 | long intergenic non-protein coding RNA 2091 | / |  |  |
| chr16:25413501:25413639_UMHL3 | 0.013 | ZKSCAN2 | zinc finger with KRAB and SCAN domains 2 | / |  |  |
| chr11:133852324:133852377_UMHL3 | 0.012 | IGSF9B | immunoglobulin superfamily member 9B | Plays a role in synapses | No | No |
| chr22:39784422:39784565_UMHL | 0.012 | TAB1 | TGF-beta activated kinase 1 (MAP3K7) binding protein 1 | Regulates PTC cells proliferation and migration / Participants in inflammatory innate immune response | Yes | Yes |
| chr20:62046303:62046323_AMF | 0.011 | CHRNA4 | cholinergic receptor nicotinic alpha 4 subunit | One of a prognostic set correlated with immune infiltration in skin cutaneous melanoma | No | Yes |
| chr9:140089655:140089861_AMF | 0.011 | TPRN | taperin | Mutations have been associated with autosomal recessive deafness | No | No |
| chr1:221051967:221051975_MHL | 0.011 | HLX | H2.0 like homeobox | Associated with the development and prognosis of Graves’ disease / Expresses in activated natural killer cells | Yes | Yes |
| chr8:38325198:38325247_AMF | 0.011 | FGFR1 | fibroblast growth factor receptor 1 | Participates in PTC proliferation and metastasis / Induce PD-L1 expression in multiple HNSCC cell lines and human immature dendritic cells | Yes | Yes |
| chr1:212687460:212687484_UMHL3 | 0.011 | LINC01740 | long intergenic non-protein coding RNA 1740 | One of three cuproptosis-associated prognostic markers of LUSC contributes to immunotherapy | No | Yes |
| chr5:15500310:15500479_MHL | 0.011 | FBXL7 | F-box and leucine rich repeat protein 7 | One gene of prognostic risk model in HCC associated with immune cell infiltration | No | Yes |
| chr20:6748842:6748856_MHL3 | 0.010 | BMP2 | bone morphogenetic protein 2 | Papillary carcinoma with intratumoral heterotopic ossification was correlated with the expression of BMP-2, leading to neovascularization / One prognostic signature in NSCLC associated with immune cell infiltration | Yes | Yes |
| chr15:27017041:27017079_UMHL3 | 0.010 | GABRB3 | gamma-aminobutyric acid type A receptor subunit beta3 | High expression with low levels of immune reactivity | No | Yes |
| chr7:30721952:30721977_PDR | 0.010 | CRHR2 | corticotropin releasing hormone receptor 2 | Expresses in both equine and bovine thyroid glands / Expresses in splenic B cells | Yes | Yes |
| chr15:44038750:44038801_MHL3 | 0.010 | PDIA3 | protein disulfide isomerase family A member 3 | Related to clinical outcomes in PTC patients / Correlated with the infiltration degree of various immune cells | Yes | Yes |
| chr1:247590087:247590125_UMHL3 | 0.009 | NLRP3 | NLR family pyrin domain containing 3 | Plays a role in the regulation of inflammation, the immune response, and apoptosis | No | Yes |
| chr1:16489135:16489154_MHL | 0.009 | EPHA2 | EPH receptor A2 | Expresses in human thyroid cancer and mediates invasion in FTC cell lines / A candidate tumor intrinsic driver of immunosuppression | Yes | Yes |
| chr4:17783433:17783483_MHL3 | 0.009 | FAM184B | family with sequence similarity 184 member B | Associated with carcass traits in Cattle | No | No |
| chr4:7474354:7474374_PDR | 0.009 | MIR4274 | microRNA 4274 | Associated with the osteoporotic phenotype | No | No |
| chr21:45160770:45160856_UMHL3 | 0.008 | PDXK | pyridoxal kinase | One gene of a prognostic cuproptosis-related model in HCC associated with immune cell infiltration | No | Yes |
| chr2:90449871:90449877_PDR | 0.007 | MIR4436A | microRNA 4436a | / |  |  |
| chr1:84326655:84326705_AMF | 0.007 | LINC01725 | long intergenic non-protein coding RNA 1725 | Participates in the wound healing process in keloid-prone individuals | No | No |
| chr22:40042775:40042781_MHL | 0.007 | CACNA1I | calcium voltage-gated channel subunit alpha1 I | Variants contribute to the risk of hemiplegic migraine | No | No |
| chr9:136357330:136357347_PDR | 0.007 | SLC2A6 | solute carrier family 2 member 6 | Monitors the immunological imprinting of macrophages | No | Yes |
| chr4:3873289:3873320_PDR | 0.006 | FAM86EP | family with sequence similarity 86 member E, pseudogene | / |  |  |
| chr8:125740528:125740563_MHL | 0.006 | MTSS1 | MTSS I-BAR domain containing 1 | Alters gravity conditions in FTC cells / Curtails LUAD immune evasion | Yes | Yes |
| chr13:26761355:26761378_UMHL3 | 0.006 | RNF6 | ring finger protein 6 | A positive mediator in the antiviral immune responses | No | Yes |
| chr1:2979941:2980204_UMHL3 | 0.005 | PRDM16-DT | PRDM16 divergent transcript | One of immune-related lncRNA Pairs in ccRCC | No | Yes |
| chr14:70346859:70346870_PDR | 0.005 | SMOC1 | SPARC related modular calcium binding 1 | Associated with tumor-infiltrating immune cells in the tumor microenvironment | No | Yes |

The feature importance is evaluated based on the weight in the random forest model. FTC, follicular thyroid cancer; PTC, papillary thyroid cancer; ATC, anaplastic thyroid cancer; HT, Hashimoto’s thyroiditis; ERAD, Endoplasmic reticulum-associated protein degradation; PDA, pancreatic ductal adenocarcinoma; CRC, colorectal cancer; HNSCC, head and neck squamous cell carcinomas; LUSC, lung squamous cell carcinoma; HCC, hepatocellular carcinoma; NSCLC, non-small cell lung cancer; LUAD, lung adenocarcinoma; ccRCC, clear cell renal cell carcinoma.

Supplementary Table 3. Clinicopathological characteristics of non-micronodules and micronodules in the study cohort.

| Characteristics | Validation cohort | | Test cohort | |
| --- | --- | --- | --- | --- |
|  | > 10mm (n=46) | ≤ 10mm (n=51) | > 10mm (n=32) | ≤ 10mm (n=56) |
| Age, years |  |  |  |  |
| Median (range) | 47 (25, 76) | 41 (25, 87) | 49 (19, 74) | 47 (17, 74) |
| Sex (n, %) |  |  |  |  |
| Male | 11 (23.9) | 11 (21.6) | 10 (31.2) | 21 (37.5) |
| Female | 35 (76.1) | 40 (78.4) | 22 (68.8) | 35 (62.5) |
| Group (n, %) |  |  |  |  |
| Benign nodule | 40 (87.0) | 15 (29.4) | 28 (87.5) | 25 (44.6) |
| Malignant nodule | 6 (13.0) | 36 (70.6) | 4 (12.5) | 31 (55.4) |
| ACR TI-RADS (n, %) |  |  |  |  |
| 1-3 | 23 (50.0) | 1 (2.0) | 19 (59.4) | 6 (10.7) |
| 4 | 16 (34.8) | 9 (17.6) | 10 (31.2) | 19 (33.9) |
| 5 | 3 (6.5) | 38 (74.5) | 3 (9.4) | 31 (55.4) |
| Not graded | 4 (8.7) | 3 (5.9) | 0 (0.0) | 0 (0.0) |
| Diameter, mm |  |  |  |  |
| Average (range) | 23.5 (8.3, 53.0) | 5.7 (3.3, 9.5) | 25.8 (8.0, 59.0) | 5.3 (2.3, 8.7) |
| Lymph node metastasis (n, %) |  |  |  |  |
| Yes | 4 (8.7) | 3 (5.9) | 0 (0.0) | 0 (0.0) |
| No | 42 (91.3) | 48 (94.1) | 0 (0.0) | 0 (0.0) |
| Methylation score |  |  |  |  |
| Median (range) | 0.46 (0.28, 0.69) | 0.54 (0.34, 0.76) | 0.44 (0.26, 0.62) | 0.51 (0.19, 0.71) |

A micronodule is defined as a nodule with a diameter of 10 mm or smaller.


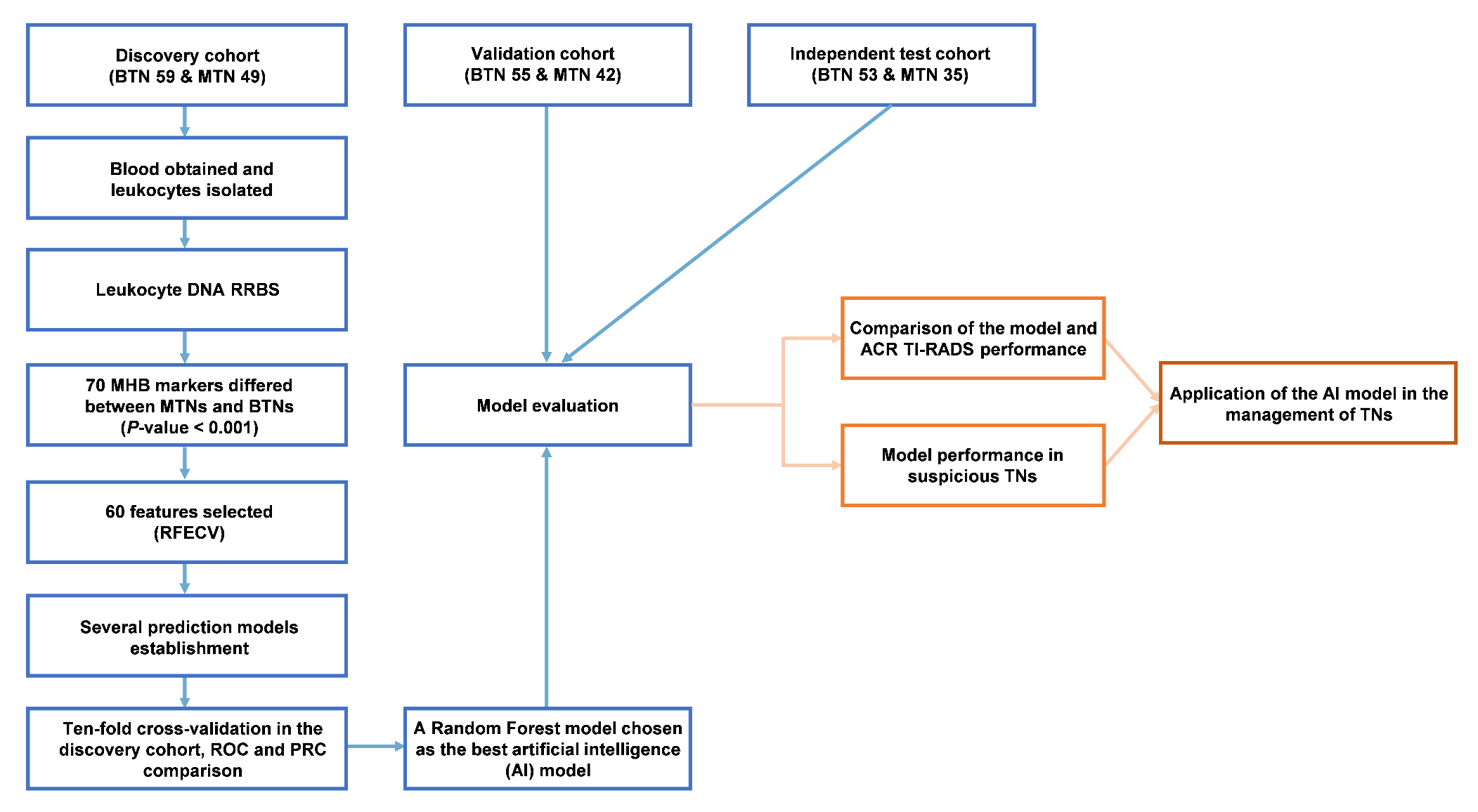


Supplementary Figure 1. Flow chart of AI model establishment. A total of 108 TN patients were enrolled as a discovery cohort. Frequently adopted models were developed and their performance was evaluated by ROC and PRC. A validation cohort consisted of 97 TN patients and an independent test cohort consisted of 88 TNs were enrolled to evaluate the performance of the best model. The samples for both the discovery and validation sets were exclusively obtained from the Department of Interventional Radiology, Zhongshan Hospital, Fudan University. Samples for the independent test cohort were collected from the Department of Ultrasound, Xinhua Hospital Affiliated to Shanghai Jiao Tong University School of Medicine. TN, thyroid nodule; BTN, benign thyroid nodule; MTN, malignant thyroid nodule; RRBS, reduced representation bisulfite sequencing; MHB, methylation haplotype block; RFECV, recursive feature elimination with cross-validation; ROC, receiver operating characteristic curve; PRC, Precision recall curve.


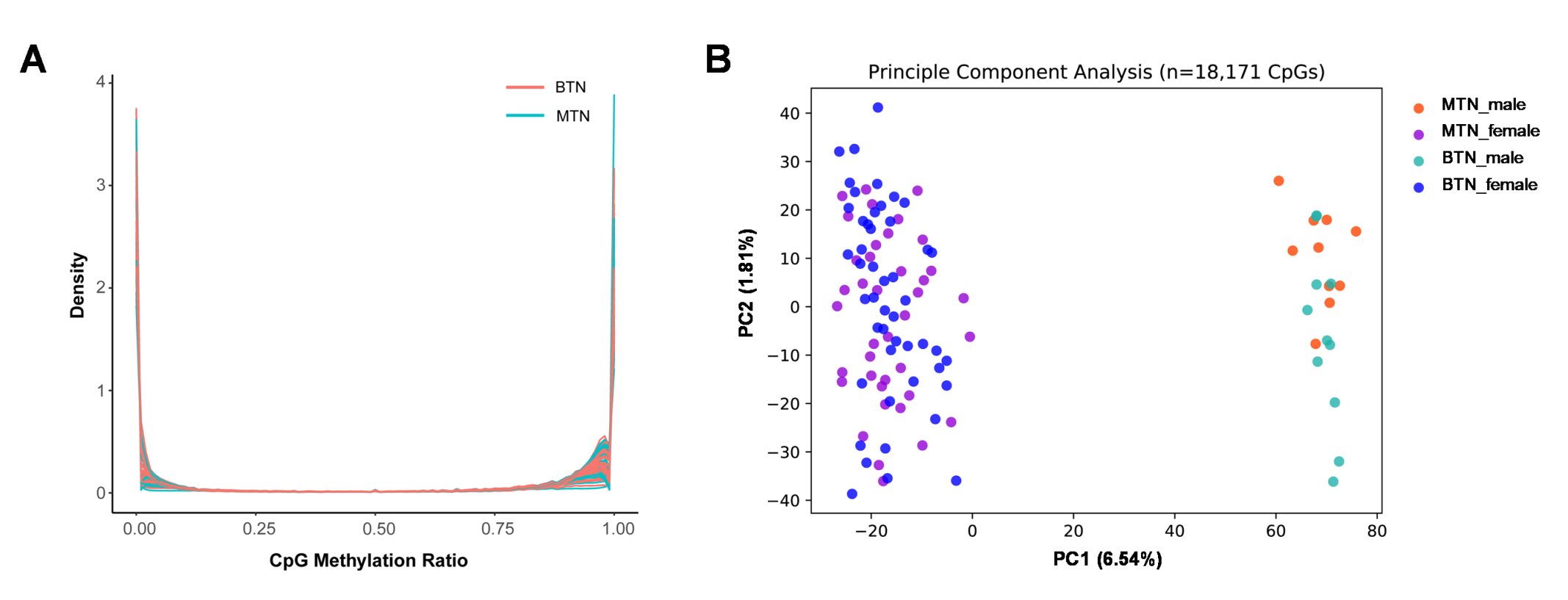


Supplementary Figure 2. Epigenetic alterations identified in the discovery cohort. CpG methylation states (A) and PCA analysis (B) across individual sequencing reads. BTN, benign thyroid nodule; MTN, malignant thyroid nodule.


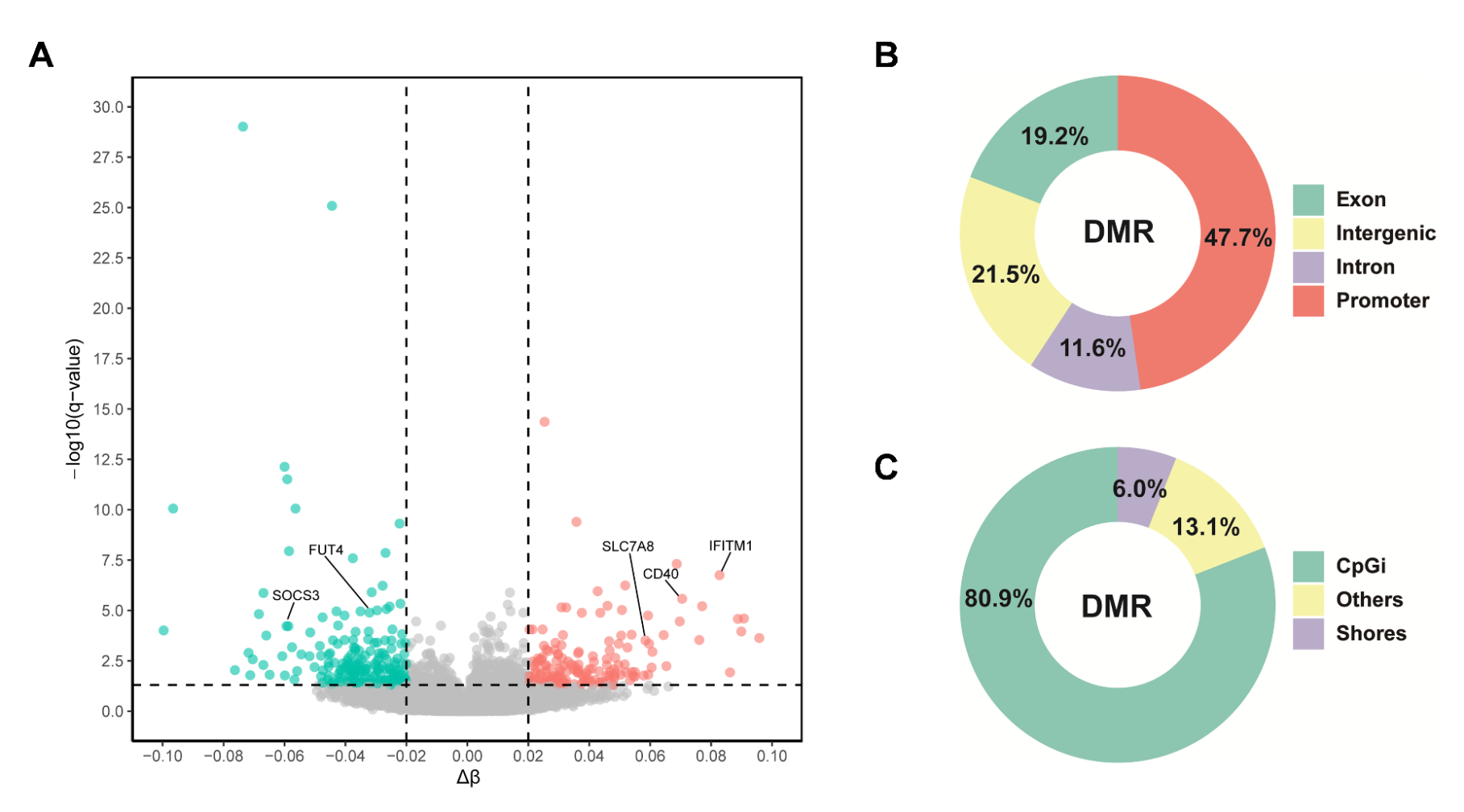


Supplementary Figure 3. Differentially methylated regions (DMRs) between MTNs (n = 59) and BTNs (n = 49). (A) Volcano plot of DMRs. Genes related to immune response are shown. Green represents hypomethylated DMRs, while red represents hypermethylated DMRs. The distributions of gene location (B) and CpG islands and their shores of these DMRs (C). BTN, benign thyroid nodule; MTN, malignant thyroid nodule.


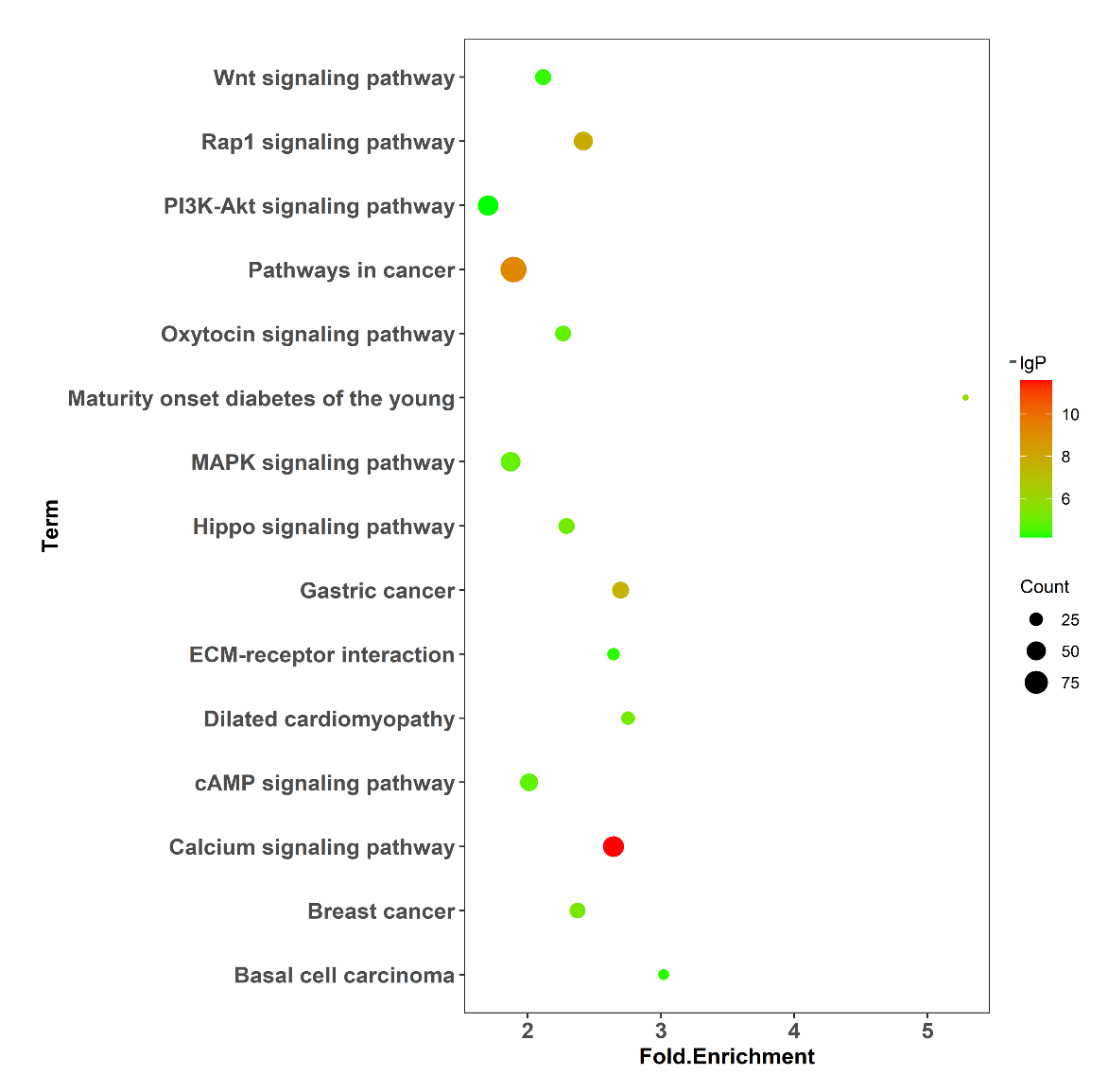


Supplementary Figure 4. KEGG pathways significantly enriched for MHBs. MHB, methylation haplotype block. - log10, - log10(*P*-value).


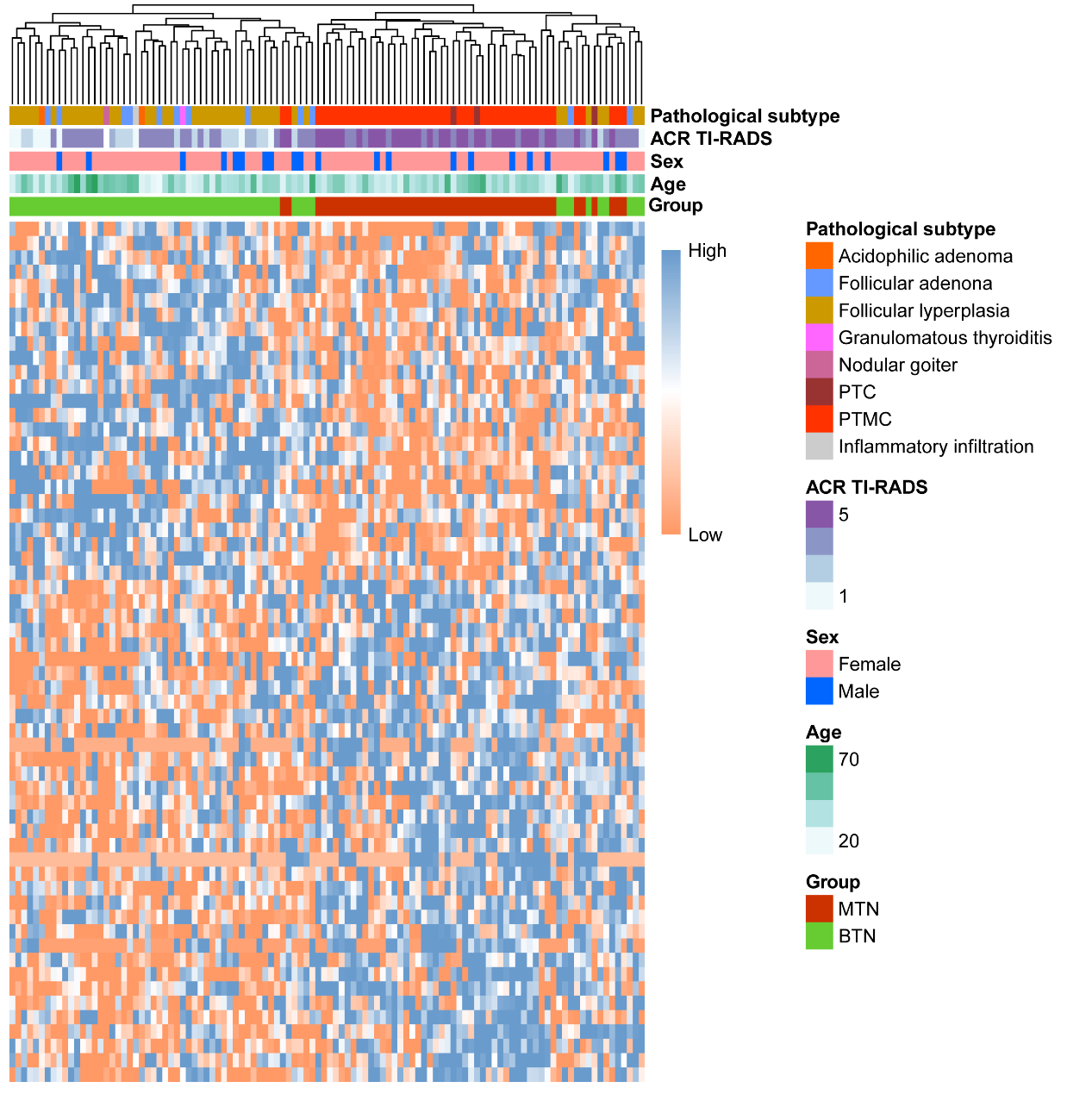


Supplementary Figure 5. A heatmap displaying 60 MHB markers involved in the model, with clinicopathological details presented on the right side. MHB, methylation haplotype block; BTN, benign thyroid nodule; MTN, malignant thyroid nodule; PTC, papillary thyroid carcinoma; PTMC, papillary thyroid microcarcinoma.


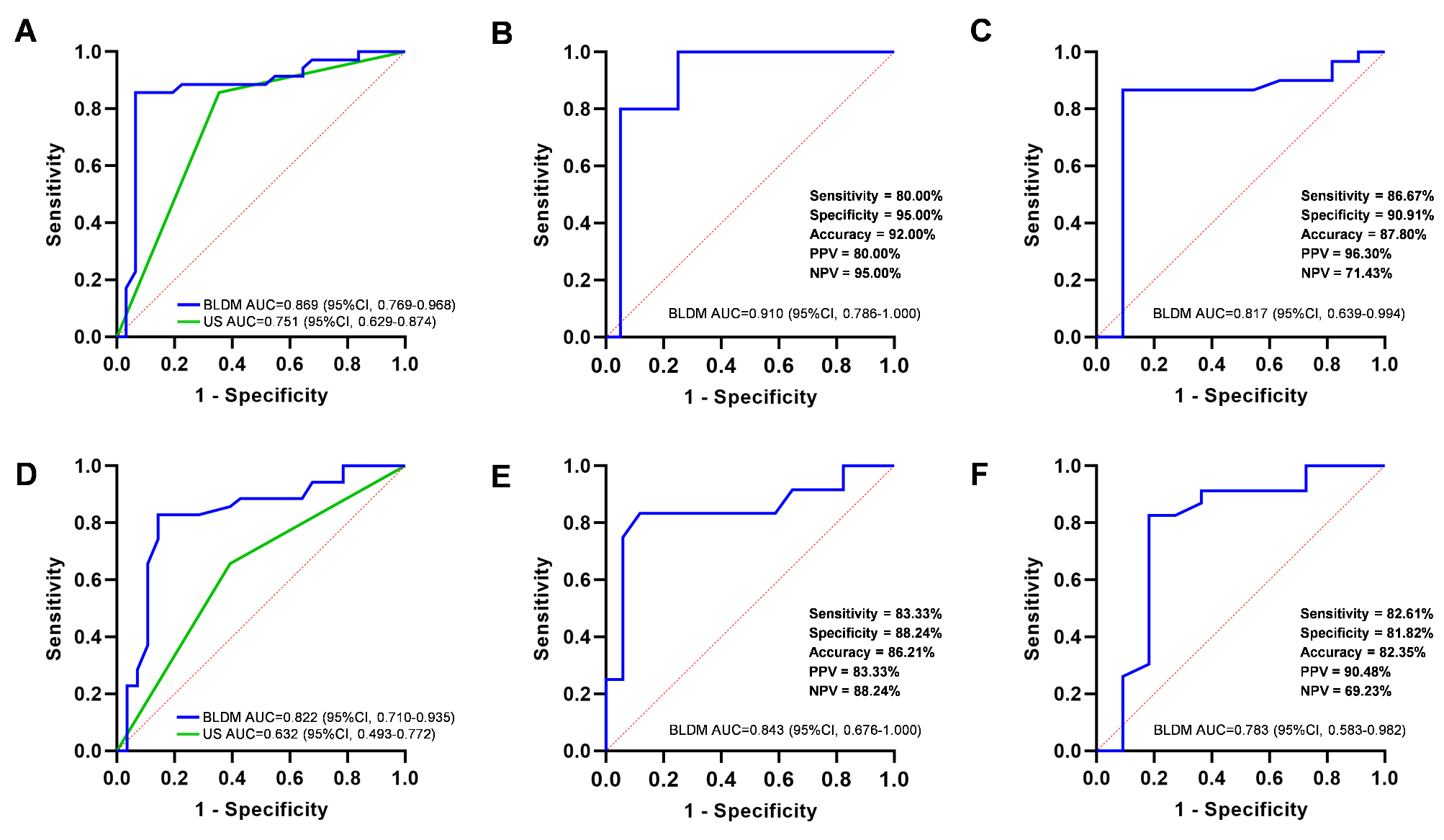


Supplementary Figure 6. Performance of the BLDM model in classifying MTN and BTN samples in ACR TI-RADS category 4 and 5. (A) The area under the curve (AUC) scores of the BLDM model and US in ACR TI-ARDS ≥ 4 in the validation cohort. (B) The AUC of the BLDM model in ACR TI-ARDS 4 in the validation cohort. (C) The AUC of the BLDM model in ACR TI-ARDS 5 in the validation cohort. (D) The AUC of the BLDM model and US in ACR TI-ARDS ≥ 4 in the independent test cohort. (E) The AUC of the BLDM model in ACR TI-ARDS 4 in the independent test cohort. (F) The AUC of the BLDM model in ACR TI-ARDS 5 in the independent test cohort. The sensitivity, specificity, accuracy, PPV and NPV of the BLDM model are shown. US, ultrasonography; PPV, positive predictive value; NPV, negative predictive value.

**
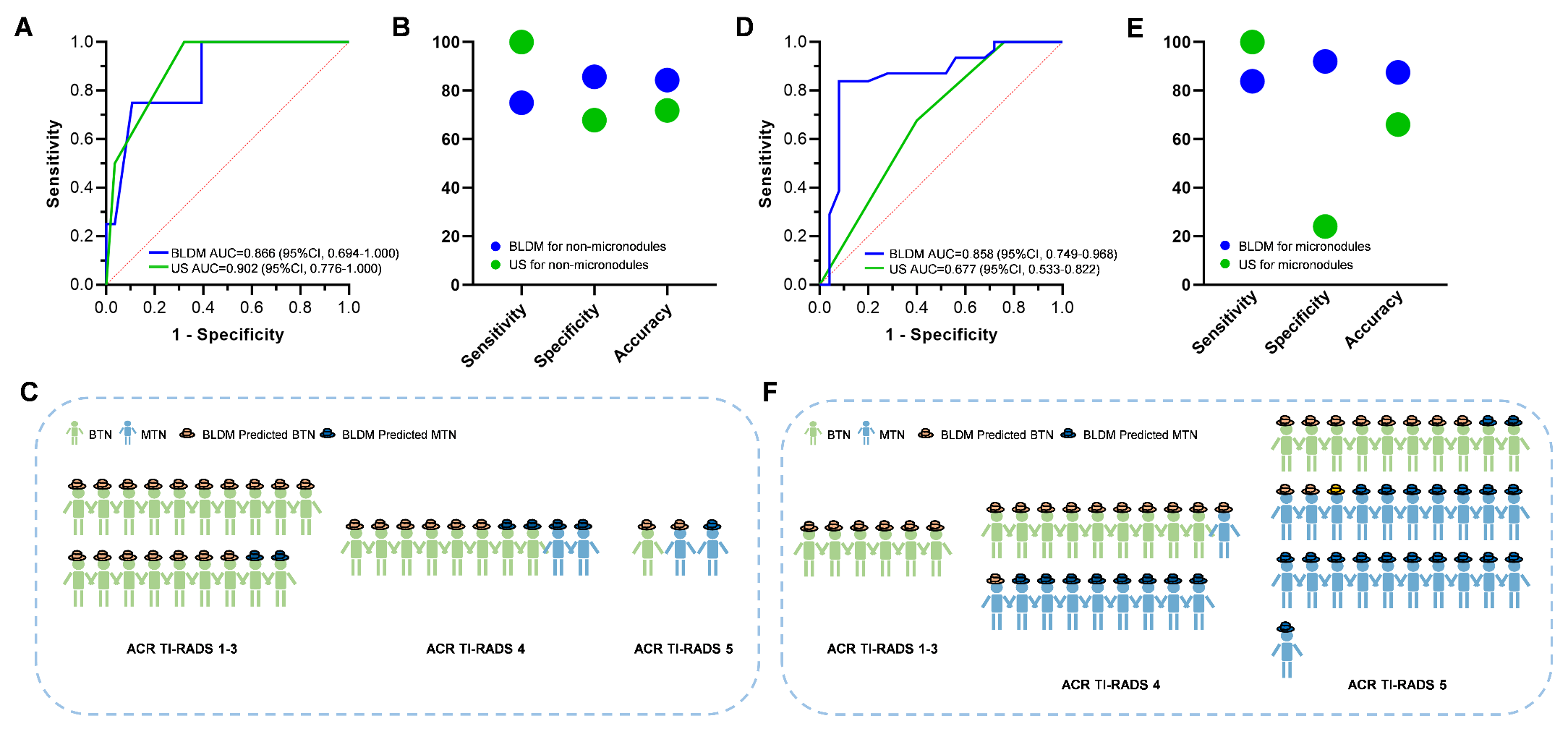
**

Supplementary Figure 7. Performance of the BLDM model in classifying MTN and BTN samples in both non-micronodules and micronodules within the independent test cohort. (A) Area under the curve (AUC) scores of the BLDM model and US for non-micronodules. (B) Comparing the performance of the BLDM model and US in non-micronodules. (C) The diagnostic performance of the BLDM model for non-micronodules across different ACT TI-RADS categories. (D) AUC scores of the BLDM model and US for micronodules. (E) Comparing the performance of the BLDM model and US in micronodules. (F) The diagnostic performance of the BLDM model for micronodules across different ACT TI-RADS categories. BTN, benign thyroid nodule; MTN, malignant thyroid nodule; US, ultrasonography.

**
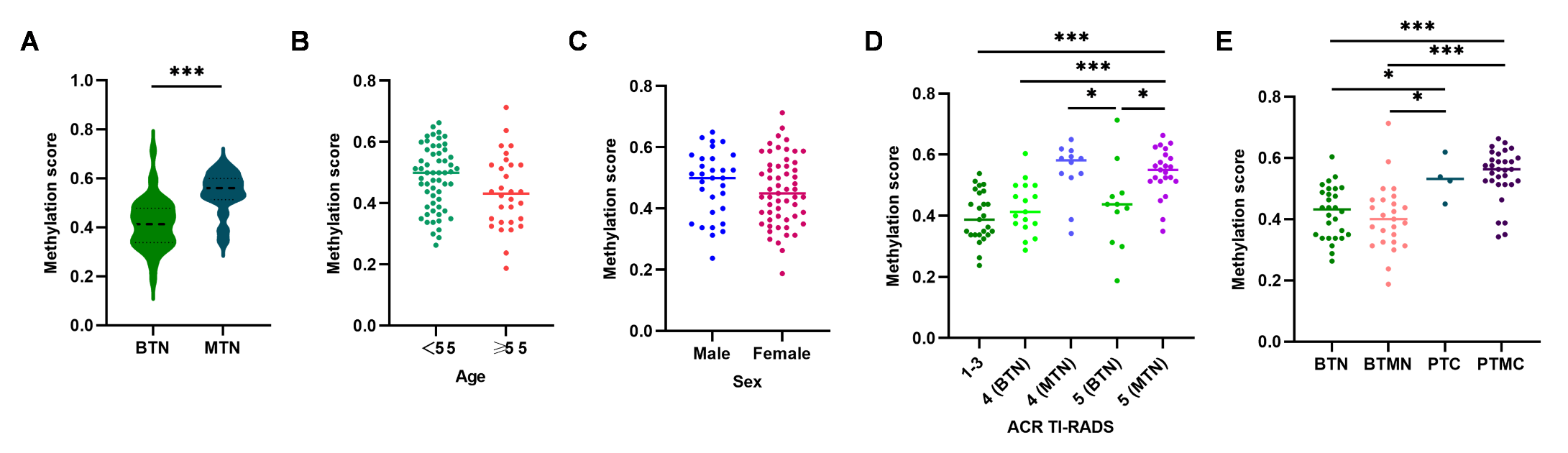
**

Supplementary Figure 8. Association of clinical features and methylation scores in the independent test cohort. The scatter plots depict methylation scores in relation to pathology (A), age (B), gender (C), ACR TI-RADS (D) and nodule sizes (E). The black horizontal line represents the median methylation levels. BTN, benign thyroid nodule; MTN, malignant thyroid nodule; BTMN, benign thyroid micronodule; PTC, papillary thyroid carcinoma; PTMC, papillary thyroid microcarcinoma. **P* < 0.05, ****P* < 0.001.
